# The Multisensory Attentional Consequences of Tool Use: A Functional Magnetic Resonance Imaging Study

**DOI:** 10.1371/journal.pone.0003502

**Published:** 2008-10-29

**Authors:** Nicholas P. Holmes, Charles Spence, Peter C. Hansen, Clare E. Mackay, Gemma A. Calvert

**Affiliations:** 1 Department of Experimental Psychology, University of Oxford, Oxford, United Kingdom; 2 Department of Psychology, Hebrew University of Jerusalem, Jerusalem, Israel; 3 School of Psychology, University of Birmingham, Birmingham, United Kingdom; 4 Department of Psychiatry, University of Oxford, and University of Oxford Centre for Clinical Magnetic Resonance Research, John Radcliffe Hospital, Headington, Oxford, United Kingdom; 5 Warwick Manufacturing Group, International Manufacturing Centre, University of Warwick, Coventry, United Kingdom; James Cook University, Australia

## Abstract

**Background:**

Tool use in humans requires that multisensory information is integrated across different locations, from objects seen to be distant from the hand, but felt indirectly at the hand via the tool. We tested the hypothesis that using a simple tool to perceive vibrotactile stimuli results in the enhanced processing of visual stimuli presented at the distal, functional part of the tool. Such a finding would be consistent with a shift of spatial attention to the location where the tool is used.

**Methodology/Principal Findings:**

We tested this hypothesis by scanning healthy human participants' brains using functional magnetic resonance imaging, while they used a simple tool to discriminate between target vibrations, accompanied by congruent or incongruent visual distractors, on the same or opposite side to the tool. The attentional hypothesis was supported: BOLD response in occipital cortex, particularly in the right hemisphere lingual gyrus, varied significantly as a function of tool position, increasing contralaterally, and decreasing ipsilaterally to the tool. Furthermore, these modulations occurred despite the fact that participants were repeatedly instructed to ignore the visual stimuli, to respond only to the vibrotactile stimuli, and to maintain visual fixation centrally. In addition, the magnitude of multisensory (visual-vibrotactile) interactions in participants' behavioural responses significantly predicted the BOLD response in occipital cortical areas that were also modulated as a function of both visual stimulus position and tool position.

**Conclusions/Significance:**

These results show that using a simple tool to locate and to perceive vibrotactile stimuli is accompanied by a shift of spatial attention to the location where the functional part of the tool is used, resulting in enhanced processing of visual stimuli at that location, and decreased processing at other locations. This was most clearly observed in the right hemisphere lingual gyrus. Such modulations of visual processing may reflect the functional importance of visuospatial information during human tool use.

## Introduction

Using a tool to act upon distant objects requires that we attend visually to the target objects and somatically to the somatosensory stimuli felt by the hand through the tool. During tool use, the brain must encode objects both visually and somatically, and integrate these multisensory inputs in the control of action. How the brain achieves this has long been of interest [1]–[2], yet the key issue regarding how the brain integrates multisensory stimuli that arise from different locations has only recently begun to be addressed [3]–[10].

During a typical study of multisensory integration and tool use, human neuropsychological patients or healthy participants hold a tool in their hand and perform a series of tool use actions. During or immediately after this period of tool use, multisensory (typically visual-tactile) stimuli are presented near to or far from the hand and tool. It has repeatedly been shown that tool use enhances the integration of multisensory stimuli presented near to the tool, as compared to stimuli presented in other regions in nearby space (for review, see [10]). Such multisensory effects have been demonstrated, for example, in neuropsychological impairments in detecting contralesional tactile stimuli in the presence of simultaneous ipsilesional visual distractors (i.e., crossmodal extinction, [3]–[6], [11]), and decreases in healthy human participants' ability to attend selectively to tactile stimuli, while trying to ignore visual distractors [7]–[9], [12]. In brief, it has been argued that, following a short period of tool use, and sometimes as soon as the tool is held [10], visual stimuli previously encoded as distant from the hand (in ‘extrapersonal’ space), may be encoded differently, as if they were actually close to the hand, in a multisensory representation of nearby ‘peripersonal’ space.

Such changes in the processing of visual and tactile stimuli have occurred following a variety of different tool use behaviours of varying complexity, from simply holding a long stick and orienting its' distal end towards a visual stimulus [13]–[14], using a stick to point to locations in space or to bisect lines [15]–[16], crossing and un-crossing two sticks or toy golf clubs, one held in each hand, over the body midline [12], [17], and to the repeated use of a rake to retrieve target objects over several minutes [4]–[6], [11]. The fact that changes in multisensory integration have been found following such simple behaviours as holding and orienting a stick towards a visual stimulus, and without the need for any prior training, has been interpreted as showing that human tool use relies, at least partly, on very rapid modifications of multisensory and sensory-motor processing in the brain [10].

One important question concerning tool use and multisensory integration remains unanswered: When we hold and use a tool, or orient its' functional part towards a particular location, where does our multisensory spatial attention go? By multisensory spatial attention, we mean ‘spatial attention that is linked across sensory modalities, and/or that has multisensory consequences’ – for example, attending to a location that is defined visually typically has consequences both for the processing of visual and non-visual stimuli presented at that location, as compared to at other locations. It seems intuitive, at least to us, that people would naturally pay more attention to the location occupied by the functional part of the tool, for this is where the crucial multisensory and sensorimotor interactions between the tool and the target object occur. Orienting one's attention to the functional part of the tool may thus result in enhanced processing of visual stimuli at that location, and perhaps also in suppressed processing of stimuli at other locations. We tested this possibility with functional magnetic resonance imaging (fMRI).

We asked young, healthy human participants to perform a simple task inside an MRI scanner. Their task was to use a long wooden rod (the ‘tool’) to locate and discriminate between two kinds of target vibrotactile stimuli (single continuous, and double pulsed vibrations) presented at the distal (functional) tip of the tool, approximately 60 cm away from the participants' hands. The vibrotactile stimuli were generated by stimulators positioned on a table over the participants' legs. Participants were instructed to ignore the simultaneously-presented visual distractor stimuli, and to make a finger button response according to the type of vibrotactile target. The visual distractor stimuli were also of two different types (continuous and pulsed), but were randomized with respect to the vibrotactile targets, resulting in one of four possible combinations of vibrotactile and visual stimuli being presented on each experimental trial. Half of these trials thus contained ‘congruent’ types of stimuli, the other half contained ‘incongruent’ types. Comparing performance between these two types of trial provided a behavioural measure of multisensory integration. It is important to note here that ‘congruence’ refers only to the relationship between the target and the distractor that is of relevance to the participants' discrimination task. In the present report, congruence refers only to the *type* of stimulus presented (continuous vs. pulsed), and *not* to the spatial location of the stimuli (see refs [18], [19] for further discussion).

This simple task was constant across all experimental conditions, for all of the participants. The participants were explicitly instructed to ignore the visual distractor stimuli, and to pay attention to, and respond only according to the type of vibrotactile stimulus (pulsed or continuous), while fixating centrally. To indicate their response, participants pressed one of two buttons using the hand opposite to the one holding the tool. At the beginning of half of the ‘rest’ blocks between blocks of the vibrotactile discrimination task, the participants were required to move the tip of the tool between one of two target locations, on the left or right of the visual and body midline. All participants trained on these tasks for at least 15 minutes prior to scanning.

**Figure 1:**
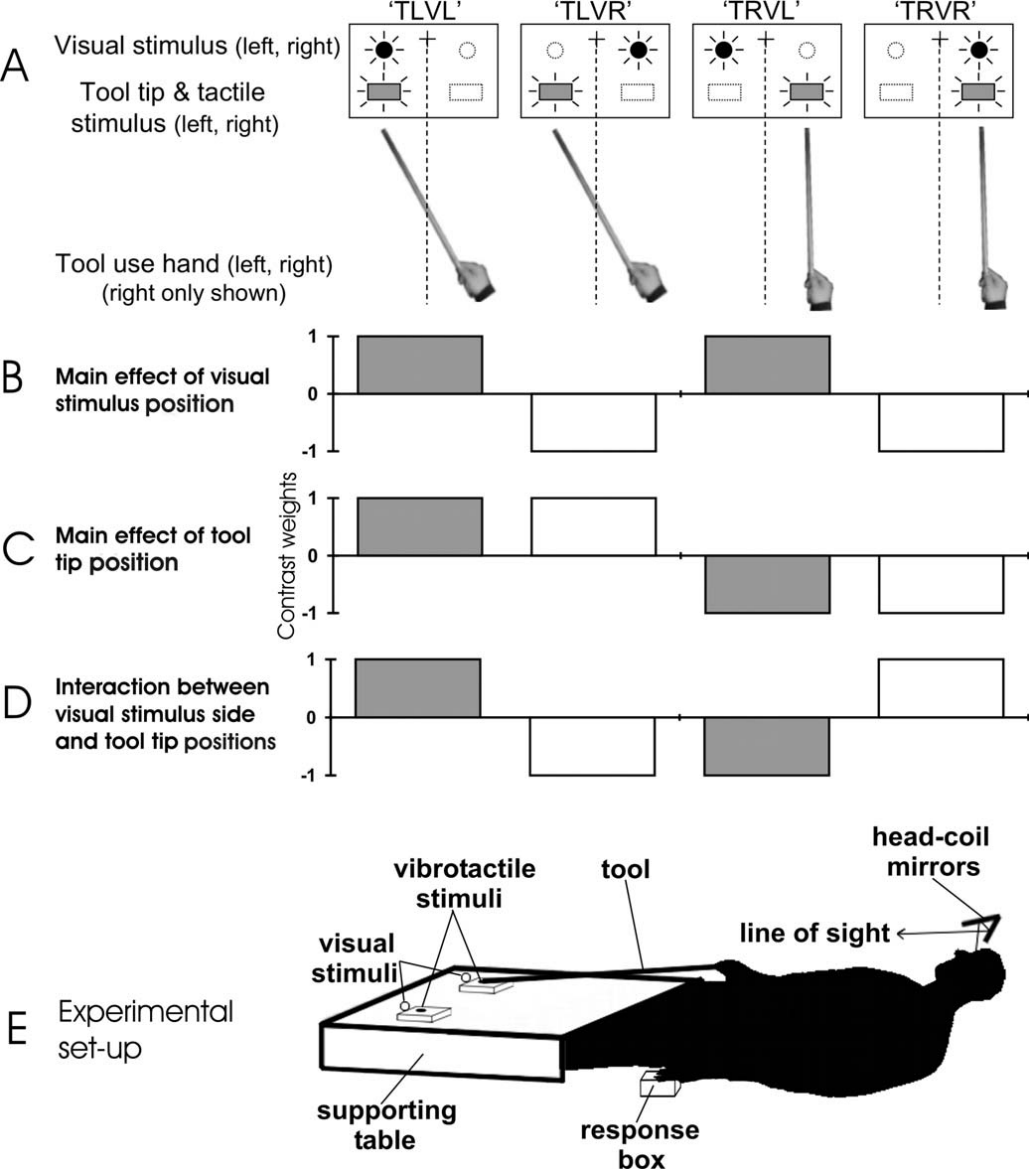
Methods and analysis. A. Four of the eight experimental conditions are depicted (i.e., for the right hand tool use experiment only). The participants held a simple tool in their hand, with the tip held on the left or the right side. Filled circles: active visual distractors in each condition. Open circles: inactive visual distractors in each condition. Filled rectangles: active vibrotactile targets in each condition. Open rectangles: inactive vibrotactile targets in each condition. B. Main effect of visual distractor position (left>right illustrated). Grey columns: left hemifield visual stimuli. White columns: right hemifield visual stimuli. C. Main effect of tool tip and vibrotactile target position (left>right illustrated). D. Interaction between visual distractor and tool tip positions (same sides>different sides illustrated). E. Experimental apparatus. The participant lay supine in the scanner bore, viewing the apparatus via a head-coil-mounted mirror system. A tool (8×750 mm wooden dowel) was held in either the participant's left or right hand, and a response box was held in their other hand. The tool was oriented towards the participant's legs. The tip of the tool was positioned on either the left or right vibrotactile target stimulator, depending on the condition, and guided by a semicircular rubber guide. The visual distractors were presented with two 10 mm red LEDs, positioned immediately above and behind each vibrotactile stimulator. The vibrotactile and visual stimulators were supported on an acrylic table, resting over the participant's legs.

There were four types of block in each run of the experiment, derived from two spatial stimulus variables: Vibrotactile target position (left or right, Figure 1C), and visual distractor position (left or right, Figure 1B). Participants performed the same two tasks in all four of these blocks – the only difference between blocks being the relative locations of the vibrotactile target (at the functional tip of the tool), and the visual distractor stimuli (Figure 1A). We performed two experiments, with 14 and 13 participants respectively, in which the only difference between the experiments was the hand that participants used to hold the tool and to perceive the vibrotactile target (i.e., right and left, respectively).

These two tasks were designed to ensure that: a) Participants were actively using the tool to perceive the vibrations; b) Participants were concentrating on task performance and fixating centrally, and; c) A behavioural measurement of multisensory integration (i.e., the effect of vibrotactile-visual distractor congruency) could be collected during the scanning session. Furthermore, the discrimination task was designed to be difficult to perform (such that participants would typically make ∼25% errors, see [9]), and required a constant maintenance of both the position of the tip of the tool, and pressure exerted on the tool by the hand, in order that the vibrotactile stimuli would be perceptible. Such tasks have frequently been interpreted within the literature as ‘active tool use’ (see [10], for review, the short discussion above, the discussion below, and [9], for further discussion), and thus provide a very simple form of tool use with which to test the hypotheses presented here.

In addition, we designed our behavioural task in order to minimize any differences in overall behavioural performance between experimental conditions (blocks) of the task. In particular, we aimed to eliminate differences in performance based on the spatial location of the visual and vibrotactile stimuli, by requiring participants to perform a non-spatial discrimination on the target vibrotactile stimuli [20]. Because the primary contrasts of interest in the analysis of the fMRI data involved comparisons between same-side and different-sides visual and vibrotactile stimulation, we aimed to ensure that there were no significant differences in behavioural performance between these conditions. Given such a difference in performance, it would be difficult to assess any effects of same-side vs. opposite-sides stimuli in the presence of the confounding effects of different reaction times and error rates, attentional load, and error-processing [21], [22]. For seven behavioural experiments investigating the subtle spatial modulations of performance that may be found when using the present task, and for further discussion see [9], [20].

If using the functional part of a tool on one side of space is accompanied by a shift of spatial attention to that side (specifically to the location of the functional part of the tool), then visual stimuli presented near the functional part of the tool should result in enhanced BOLD response in retinotopic portions of occipital visual cortex (because they are attended), as compared to the exact same stimuli presented when the tool is positioned elsewhere (because these stimuli are now unattended) [21], [23]–[26]. Alternatively, if participants are able completely to ignore the visual distractor stimuli, as they were explicitly instructed to do, then there should be no effect of stimulus congruency (i.e., no multisensory integration), and, furthermore, no effect of the position of the tool on the processing of visual stimuli in occipital cortex. We tested these two alternatives in a two-stage thresholding procedure [27]. First, we defined a volume of interest based upon the two simple effects of visual distractor location: left>right distractors, and right>left distractors. Based on previous results and the well-known functional organization of the visual brain, we expected that two clusters of activation would result from these contrasts - primarily in occipital cortex contralateral to the visual distractors. Next, we searched within these volumes for brain areas in which the BOLD response varied significantly as a function of the position of the functional part of the tool, with the prediction that a shift of spatial attention to the functional part of the tool should result in increased BOLD response related to the visual distractor stimuli in the contralateral occipital cortex, and decreased BOLD response in the ipsilateral occipital cortex [21]. The resulting clusters would therefore reveal voxels that showed both a significant main effect of visual distractor position, *and* a significant influence of tool position on the processing of the visual distractors.

In additional analyses, we examined the possibility that the visual and vibrotactile stimuli may activate a ‘hand-centred’ or ‘tool-centred’ multisensory brain area [10], we examined the functional and effective connectivity of one occipital area that showed clear tool-position-dependent modulation of BOLD responses, and we also examined the possibility that the behavioural measurement of multisensory interaction would significantly predict the BOLD response in visual, somatosensory, multisensory, or general attention- or response-related brain regions. Finally, we combined the above two analyses in order to search for brain regions which showed both a main effect of visual stimulus position within-participants, and a significant covariation between BOLD and our behavioural measures of multisensory integration between-participants.

## Results

### Behaviour

During the scanning sessions, there were large and significant behavioural effects of multisensory congruency (i.e., the difference in RT and errors between trials with incongruent and congruent stimuli, where congruency is defined solely by the type (single vs. double) of vibrotactile and visual stimuli). These multisensory integration (MSI) effects were present in multivariate measures combining RT and error scores (F(2,10) = 21.03, p = .0003), as well as separately for RT (F(1,11) = 45.28, p = .00003, mean±s.e.m., MSI = 78±12 ms), and errors (F(1,11) = 14.06, p = .003, MSI = 12±3%, see [9]). These behavioural results demonstrate that strong multisensory interactions occurred during acquisition of the fMRI data (Figure S1). These congruency effects were slightly larger in general when the tool was held on the right, as compared to on the left, side of visual fixation (p = .013), irrespective of which hand held the tool, and the location of the visual distractor.

As intended, the position of the visual distractor did not significantly affect behavioural performance, allowing us to rule out simple behavioural differences as potential explanations for differences in brain activation in the following fMRI contrasts of interest (see also Text S1, Figure S1, and [9], [20], for additional analyses of behavioural data). The absence of a significant effect of visual distractor position (i.e., a spatial variable) is not surprising – when participants perform a non-spatial discrimination task, it is actually quite rare to find modulations in performance based upon the spatial locations of the stimuli, provided that adequate control over the effects of stimulus-response compatibility is achieved (see [20], for further discussion and references). Nevertheless, using the present discrimination task, it is still possible to uncover very subtle spatial modulations in performance, given a sufficient sample size (e.g., n = 24), a good psychophysical environment, and when responses are executed with the feet [9], [20]. Indeed, the task used in the present study was designed explicitly, over these seven previous experiments, to minimize any differences in RT and error performance with respect to stimulus location, while allowing the use of single hand-held tool inside the MRI scanner. If RT and error performance had been significantly and strongly affected by spatial aspects of the behavioural task, it would have been very difficult for us subsequently to argue that any changes in BOLD response between conditions with different spatial configurations of target and distractor were not simply due to general differences in response speed, arousal, vigilance, or error-related processing. By successfully eliminating gross differences in behavioural performance, we can proceed to interpret changes in the BOLD response more clearly with respect to the experimental variables of most interest – i.e., the spatially-selective influence of the functional part of a tool on visual processing. For seven behavioural experiments (total n = 196) investigating and discussing these issues in detail, see [9], [20].

### fMRI

The fMRI BOLD data were initially analyzed in three ways: First, we analyzed the within-participant mean BOLD signal changes using contrasts specifying the main factors of the hand used, tool tip and vibrotactile target position, and visual distractor position, according to the models presented in Figure 1. Second, we analyzed the between-participant covariation in the magnitude of multisensory integration (as measured with the behavioural responses), and the magnitude of BOLD signal change. Third, we repeated the first analysis, this time restricting the search volume to only those brain regions showing significant activations or deactivations in the second set of (multisensory) analyses. The first two analyses were performed initially across the whole brain: Parametric maps of the Z-statistic were thresholded at Z≥2.33 (p≤.01), and the size of resulting clusters of activation were assessed for significance against Gaussian Random Field theory, resulting in a final whole-brain corrected cluster significance of p≤.05.

### Effects of visual distractor position and tool tip position

The main effect of visual distractor position, as expected, resulted in two large clusters of activation, both covering the dorsal and ventral occipital cortex contralateral to the side of the visual distractor (i.e., right occipital cortex was significantly more active for visual stimuli to the left of fixation than for right-side visual stimuli, and vice versa for left hemisphere occipital cortex). To test the potential modulatory effects of tool position, we further searched within each contralateral occipital cluster for regions that showed tool tip position-dependent modulations of BOLD response (Z≥2.33, p≤.01, voxelwise uncorrected, [27]). This analysis revealed those regions which, for example, showed a higher BOLD response to visual stimuli presented on the left as compared to on the right of fixation (the main effect of visual distractor position), but *also*, and critically, showed a higher response to the same left visual distractor when the tip of the tool was used next to it on the left, as compared to when it was used on the opposite side (i.e., the effect of tool tip and/or vibrotactile target position). In the following analyses, it is important to note that, in testing the visual and tool tip position effects, the contribution of the hand that holds the tool is effectively ignored - these analyses were performed by collapsing across the variable hand, and in any case the BOLD signal changes of interest were independent of which hand held the tool (data reviewed but not shown). Furthermore, note that the tool was present and remained stationary for at least 12 s (and, for half of the blocks, 36 s) before the start of each experimental block – this aspect of the design ensured that all activations and deactivations which were time-locked to a block of trials, reflected significant changes with respect to a baseline in which the tool was also present. In short, the presence of the tool, as an additional visual stimulus, cannot have been responsible for modulating the BOLD signal recorded during the task blocks. The possibility that these two independent visual stimuli (the distractor and the tip of the tool) in the same region of the visual field led to an interaction or non-linear supra-additive summation, of BOLD responses is considered in detail in the Discussion.

**Figure 2:**
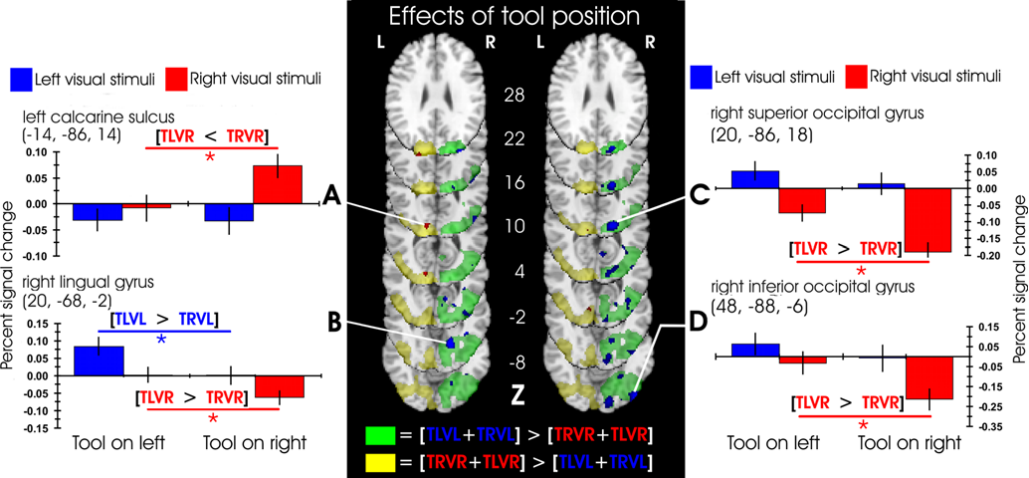
Effects of visual distractor position and tool tip position. The central panel shows the significant clusters of activation for the simple effects of visual distractor position, and the effects of tool tip position within those visual contrasts, overlaid on a standard brain in MNI template space. Green and yellow clusters show the simple main effects of visual distractor position (green: left>right; yellow: right>left), in the right and left hemispheres respectively. Within each of these clusters, specific activation peaks are highlighted in blue (tool-mediated increases or decreases in the right hemisphere) and red (tool-mediated increases or decreases in the left hemisphere). These specific activation peaks showed significant (Z≥2.33, p<.01, uncorrected) positive (contralateral increases, examples in the left data panel) or negative (ipsilateral decreases, examples in the right data panel) differences between either of the individual contrasts [TLVL>TRVL] or [TRVR>TLVR], thresholded at Z≥1.96, for display. The left and right data panels show mean±s.e. percentage BOLD signal change across the indicated peak and 4–20 neighbouring voxels. Activations related to the left visual distractors are shown in blue, and to the right visual distractors in red. L: left hemisphere. R: right hemisphere. Numbers in parentheses next to the graphs, and in the centre of the central panel, show MNI standard template coordinates in mm.

Within the clusters identified by the main effects of visual distractor position, several areas in occipital cortex showed tool tip position-dependent modulation of BOLD response, evidenced as both increases and decreases in BOLD signal with respect to the baseline. Significant *increases* in BOLD signal were found in occipital cortex *contralateral* to the visual distractor when the tip of the tool was used on the same side of fixation as the visual distractor (as compared to when it was used on the opposite side). Conversely, significant *decreases* in the BOLD response were observed in the occipital cortex *ipsilateral* to the visual distractor, when the tip of the tool was used on the opposite side of fixation to the visual distractor (as compared to on the same side). These data are shown in Figure 2A–D, Table S1, and as detailed below.

#### Right occipital cortex activations

Using the tip of the tool to perceive vibrotactile stimuli on the left of fixation (as compared to on the right) increased the BOLD response resulting from a left visual stimulus, in a portion of the right lingual gyrus (peak Z-statistic = 3.55, (20, −68, −2), Figure 2B), in an area that probably lies close to the border of retinotopic visual areas VP, V2, and V4v, representing the horizontal meridian in the left visual field, where the visual stimuli were presented [24], [28]. This region has previously been activated in tasks involving visual spatial attention [25], and by the selection of visual and tactile targets for eye movements [29]. This cluster of right hemisphere lingual gyrus voxels also showed a significant *decrease* in BOLD response for right visual stimuli when the tip of the tool was used on the right side of fixation (as compared to when used on the left, see below). Additional tool tip position-dependent increases in BOLD signal following right visual stimuli were observed in the right posterior intraparietal sulcus and the superior (Z = 2.50, (32, −80, 40)), and inferior divisions of lateral occipital cortex (Z = 2.34, (50, −78, 16)). A nearby site in the superior division of the lateral occipital cortex (24, −82, 38) was previously shown to be activated in a task-independent fashion by spatially-congruent visual and vibrotactile stimulation [30]. The superior lateral occipital region activated in the present study is close to a region activated more strongly by spatial than by orientation visual discriminations [26].

#### Left occipital cortex activations

When the tool tip was used to perceive vibrotactile stimuli on the right side of fixation, with a concurrent right visual distractor (as compared to on the left side, away from the same stimulus), the BOLD response in left occipital cortex was enhanced, with the peak voxel lying in primary visual cortex (Z = 2.87, (−14, −86, 14), Figure 2A), according to probabilistic cytoarchitecture [31]. This portion of visual cortex has previously been activated during saccades to visual and tactile targets [32], and has been argued to show supra-additive summation of visual and auditory inputs [33], [though see 27], suggesting a significant role for primary and secondary visual cortices in a number of multisensory integration tasks.

#### Right occipital cortex deactivations

Tool tip position-dependent *decreases* in BOLD response were also observed in the right occipital cortex. Activity related to visual stimuli presented on the right of fixation, in the right superior and inferior divisions of lateral occipital cortex (Z = −3.57, (20, −86, 18), Figure 2C; Z = −2.66, (48, −88, −6), Figure 2D), probably the dorsal portion of V3 [28], [34], and in the right occipital pole (Z = −2.34, (14, −92, −8)), probably comprising part of visual area V2 [34], was significantly lower when the tool tip was used on the right side of fixation as compared to on the left.

#### Tool-position-dependent increases and decreases in BOLD response within the same voxels

We noticed that a region of the right hemisphere lingual gyrus, as well as a number of other areas, displayed both increases and decreases in BOLD response relative to baseline, depending on tool position. We therefore performed an additional contrast, which assessed tool-position-dependent increases and decreases simultaneously. This contrast searched for areas in which the effect of visual stimulus position (either left>right or right>left) was larger when the tip of the tool was present next to the visual stimuli as compared to the effect of visual stimulus position when the tool was on the opposite side (i.e., for left>right visual stimuli: [TLVL>TRVR]>[TRVL>TLVR], and similarly for right>left visual stimuli). This contrast was assessed (voxelwise, p<.05, Bonferroni corrected) within a search volume restricted by a number of functionally-relevant criteria. We restricted the search volume to those voxels which: a) Showed a significant main effect of visual stimulus side (voxelwise Z≥2.33, p<.01, whole-brain cluster corrected to p<.05); b) Showed an increased response (Z>0, p<.5, uncorrected) when the tool was next to the contralateral visual distractor as compared to when positioned ipsilaterally (e.g., [TLVL>TRVL]), and; c) Showed a decreased response (Z<0, p<.5, uncorrected) when the tool was next to the ipsilateral visual distractor as compared to when positioned contralaterally (e.g., [TRVL>TRVR]). This multi-stage masking procedure resulted in a search volume of 6,409 voxels in the right hemisphere occipital cortex, and 3,118 voxels in the left hemisphere occipital cortex. The only brain region to survive these stringent multiple statistical criteria was a cluster of 13 voxels in the right hemisphere lingual gyrus, with peak Z-statistic of 3.93 and MNI coordinates: (18, −66, −2).

In summary, the analysis of the effects of tool position on the spatial processing of visual stimuli revealed clusters of activation contralateral to the visual stimuli, and clusters of deactivation ipsilateral to the visual stimuli. Of most prominence, a region in the ventral occipital cortex, most likely at the border of areas V2 and VP, showed both contralateral activations, and ipsilateral deactivations, depending on the relative positions of the tip of the tool and the visual distractors. Activity in this area of the right lingual gyrus showed clear modulations of visual processing during tool use. In analyses reported below, this area will also be shown to modulate its activity significantly as a function of the behavioural measures of multisensory integration, both in RT and percentage error measurements.

Since the BOLD response in the right lingual gyrus was sensitive to the position of the tool relative to the visual distractor on both sides of space, and also varied as a function of the behavioural measures of multisensory integration, we further analysed the ‘functional-’ and ‘effective-connectivity’ of this area. The raw signal, and the raw signal multiplied by +1 for task-, and −1 for rest-related epochs respectively, was entered as a regressor for each participant and run separately. Group analyses (voxelwise threshold of Z≥3.09, p≤.001, whole-brain cluster corrected, p≤.05) revealed that BOLD signal in widespread regions of bilateral dorsal, ventral, and lateral occipital cortex, bilateral superior and middle temporal gyri, and bilateral insula, thalamus, and putamen, covaried with signal in the right lingual gyrus (‘functional connectivity’). This large cortical territory may in part reflect vascular or other processes of little interest, so we further assessed the ‘effective connectivity’ of the right lingual gyrus. Signal covariation that was stronger during task periods than rest periods was restricted to bilateral portions of the occipital lingual and fusiform gyri, bilateral intracalcarine sulcus, and lateral occipital cortex in both hemispheres. These analyses suggest that, during the task periods, the right lingual gyrus functioned together with numerous regions in bilateral occipital cortex.

In an additional analysis, we searched for brain regions which showed a significant interaction between the positions of the visual distractor and the tip of the tool, both for the two hands (experiments) together, for each hand (experiment) separately, and for the three-way interaction including the factor of which hand held the tool (experiment). Such regions might be predicted to exist based on the idea of hand-centred or tool-centred processing of multisensory stimuli during tool use [10]. No such regions were found in the present datasets, either when searching across the whole brain, or restricting the search volume to just frontal and parietal sensory-motor areas (defined operationally as the approximate borders of Brodmann's areas 5, 6, 7, 8, 39, 40, 44, and 45, by creating a mask in MRICro, using canonical Brodmann area maps, overlaid in standard MNI space and smoothed with a 3D isotropic 4 mm FWHM filter to accommodate anatomical variability and imprecision). For comparison with the principal analyses reported in the present manuscript, uncorrected peak voxel coordinates and statistics for the interaction effects are provided in Table S2. Possible reasons for this failure to find significant hand- or tool-centred activations are discussed below. All other potentially relevant contrasts were examined within the factorial design of our experiments, for example, the simple effects of hand used, and the interaction between hand used and tool position. None of these additional contrasts resulted in any significant clusters of activation, and were not, in any case, of interest in the present report, so are not detailed further.

### Covariation of BOLD with behavioural measure of multisensory integration

In the second set of analyses, we used the behavioural measurements to search for activity reflecting processes of multisensory integration. Mean RT and percentage error scores were calculated for each participant and for each of the eight conditions, the across-participant mean was subtracted from each score, and the scores were then entered as predictors in whole-brain analyses separately for RT and error scores. Contrasts were specified to highlight activity that significantly covaried (either positively or negatively) with the behavioural measures of multisensory integration.

**Figure 3:**
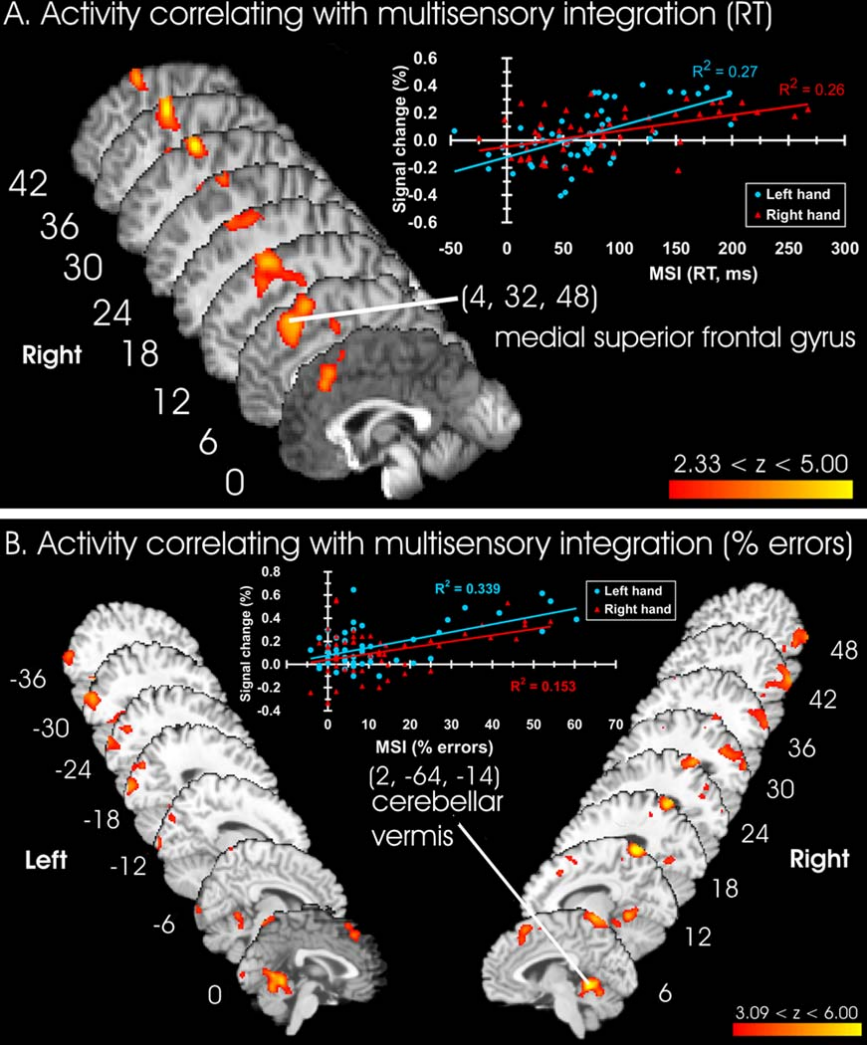
Activity positively correlated with multisensory integration. A: RT measures; B: % error measures. Clusters of activation show brain areas in which the BOLD response significantly covaried with the magnitude of multisensory integration across participants, overlaid on a standard brain in MNI space. The example data plots show the mean percentage signal change, per condition and participant, of the indicated peak and 5–20 neighboring voxels as compared to baseline (y-axis) against the magnitude of multisensory integration derived from behavioural measurements (x-axis). For display purposes, data were pooled for the left hand (blue circles) and the right hand (red triangles). MSI: multisensory integration. The threshold was set at Z≥2.33, p≤.01 for RT, and Z≥3.09, p≤.001 for errors, for display purposes only.

Multisensory integration, as indexed by RT measurements, covaried positively with BOLD response in the right hemisphere middle and superior frontal gyri (pre-frontal eye field (pre-FEF) and dorsal premotor cortex, Z = 5.48, (32, 10, 62), pre-supplementary motor area (pre-SMA), Z = 4.40, (12, 24, 60), extending medially and inferiorly into the medial surface of the superior frontal gyrus (Z = 4.59, (4, 32, 48), Figure 3A), and the border of the anterior cingulate (Z = 3.51, (8, 0, 46)). A full list of peak voxel coordinates is provided in Table S3. A similar analysis for the percentage of errors revealed four clusters of activation (Figure 3B and Table S3). One cluster overlapped considerably with the activation related to RTs in the superior and medial frontal gyrus. This overlap was not unexpected, given the high correlation between RT and error scores (left hand tool use, r(12) = .83, right hand tool use, r(12) = .62, both p≤.05). Together, these activations in dorsal premotor cortex, pre-FEF, pre-SMA, and the anterior cingulate likely reflect between-participants variation in neural processing related to response selection, the resolution of response conflicts, and the error-related processing that would frequently occur on incongruent trials [35]–[36].

The cerebellar vermis showed the strongest correlation with percentage errors (Z = 6.78, (8, −52, −6)), and this activation extended into the right cerebellar hemisphere (Z = 4.35, (18, −36, −22), Figure 3B). These activations likely reflect additional sensorimotor components of multisensory task performance. The largest regions of activation were found in occipital cortex bilaterally, including the inferior and superior divisions of lateral occipital cortex, and extending into the middle temporal gyrus, precuneus, and parietal-occipital sulcus. These large clusters included activation of both primary and secondary visual cortex (peak Z = 4.84, (−18, −98, 10)), along with bilateral regions of extrastriate cortex. Nearby activation peaks have been reported in association with attending to vision over touch [32], [37]–[39], shifting compared to holding visual attention [40], and in visual-haptic priming [41]. Several of these regions lie close to the region identified as the lateral occipital tactile-visual area by Amedi and his colleagues (LOtv, [42]).

In summary, activity in midline and lateral cerebellum, right hemisphere medial and superior frontal cortex, and lateral occipital cortex covaried significantly and positively with the behavioural measures of multisensory integration. A number of other regions showed significantly negative covariation with behavioural performance, and may relate to ‘default network’ activity (see Text S1, Table S4, and Figure S2). Together, activity in the regions identified by the behavioural covariate contrasts explained a large proportion of the variance in the behavioural measures. These regions are likely to be responsible for directing attention to touch in the presence of visual distractors, to the integration of the multisensory stimuli, for detecting and resolving the response conflicts associated with selecting responses in the presence of incongruent multisensory cues, and for error-related processing.

### Reference frames for multisensory integration

We performed three further analyses, combining the two different sets of analyses reported above. First, we examined the peak Z-statistics for the voxels identified by the simple effects of visual distractor position, tool tip position, hand used, and their interactions, as reported above (see Table S1 & Table S2). Of the nine peak voxels identified (from non-significant clusters within the ‘sensory-motor’ volume of interest described above, with a voxelwise uncorrected Z≥2.33, p≤.01) in the interaction between visual distractor position and tool tip position, only one of these voxels also showed a significant effect (voxelwise uncorrected Z≥2.33, p≤.01) in the multisensory behavioural covariate (RT) contrast. This voxel was located in the middle frontal gyrus, anterior to the frontal eye fields ((30, 12, 60), ‘pre-FEF’ [43]). Conversely, of the seven peak voxels identified by the simple effects of tool position within the significant clusters defined by the simple effects of visual distractor positions, three voxels showed significant positive effects in both the multisensory (RT) and the multisensory (error) contrasts (in right primary, secondary, and extrastriate visual cortices, (14, −92, −8), (20, −68, −2), (44, −84, −6)).

**Figure 4:**
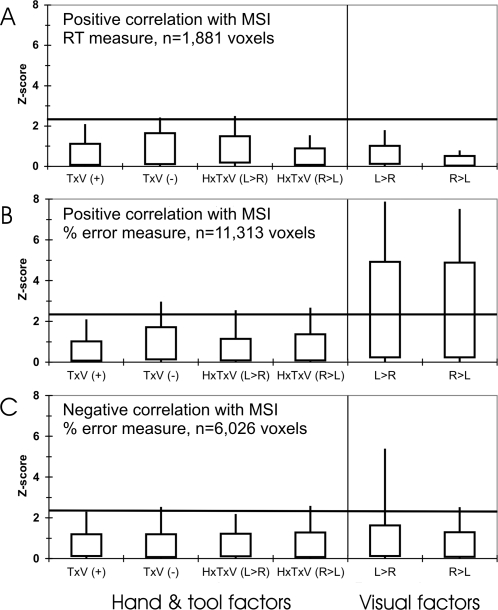
Z-statistic distributions for the main effects, simple effects, and interactions. These plots show only those voxels in which the BOLD response covaried significantly positively or negatively with the behavioural measures of multisensory integration. Boxes: 10–90% ranges. Whiskers: 0–100% ranges. A: Positive correlation with reaction times. B: Positive correlation with % errors. C: Negative correlation with % errors. Each voxel contributes 9 data points, one to each of 3 pairs of contrasts along the category (x-) axis for each of the three panels. T: main effect of tool tip position. V: main effect of visual distractor position. H: main effect of hand used. TxV(+): Voxels with a positive Z-statistic for the tool tip position x visual distractor position interaction. TxV(−): Voxels with negative Z-statistic for this interaction. HxVxT(L>R): Voxels with larger Z-statistic for the TxV interaction when using the left hand than the right hand. HxVxT(R>L): Voxels with larger Z-statistic for the TxV interaction when using the right hand than the left hand. L>R: Main effect of visual distractor position (left side>right side). R>L: Main effect of visual distractor position (right side>left side). Horizontal lines: Statistical threshold (Z≥2.33, p≤.01, voxelwise uncorrected) used in the contrasts.

Second, we examined the distributions of Z-statistics across all the voxels identified by the three multisensory contrasts (i.e., the contrasts identifying positive correlations with RT and errors, and the negative correlation with errors, Figure 4). The population of voxels positively correlated with multisensory integration (errors) showed a wide distribution of Z-statistics for the simple visual effects, from −7.51≤Z≤7.87, with mean positive and negative Z-statistics of 2.15, and −2.39, respectively for the VL>VR and VR>VL contrasts. These data indicate a strong shift in the distribution towards a significant preference for contralateral over ipsilateral visual stimuli, across the population of 11,313 voxels. Conversely, the range of Z-statistics for the interactions between visual distractor position and tool tip position was much reduced (−2.97≤Z≤2.55), and the mean Z-statistics for the four relevant contrasts were +0.50, +0.52, and −0.63, −0.83, respectively. Neither the positive correlation with RTs, nor the negative correlation with percentage errors, showed any strong trends in any contrasts.

**Figure 5:**
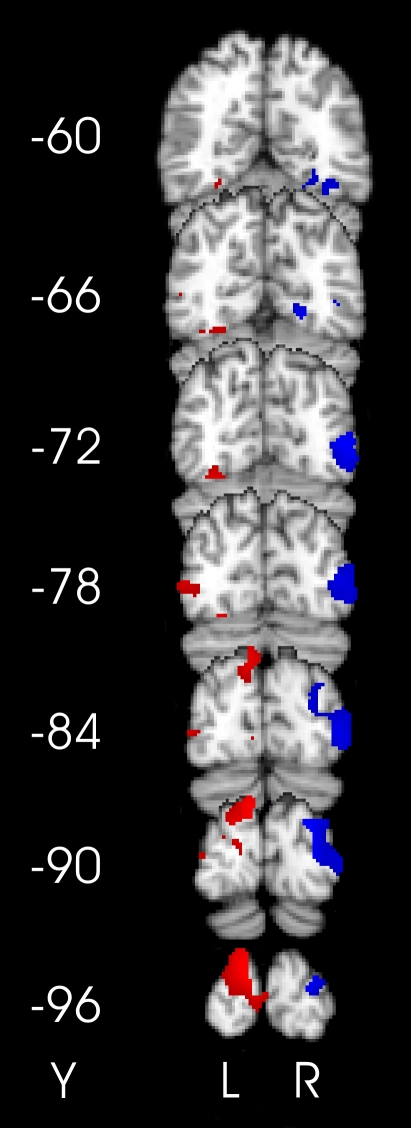
Main effects of visual distractor position within clusters defined by the multisensory contrasts. Activity significantly correlated with multisensory integration (either RT or % error measures), which also showed a significantly higher response for contralateral versus ipsilateral visual distractors. Red clusters show left hemisphere occipital regions showing both higher activation for right than for left visual distractors, and a significant covariation of BOLD signal change with the behavioural measures of multisensory integration. Blue clusters show similar regions in the right occipital cortex with a preference for left over right visual stimulation, and a significant covariation of BOLD with the behavioural measures of multisensory integration. Voxels were thresholded at p≤.05, voxelwise Bonferroni-corrected across the volume defined by the 19,220 voxels showing a significant covariation with the behavioural measures of multisensory integration.

Third, and more formally testing the above two analyses, we re-assessed the main effects, simple effects, and interactions reported in the very first set of analyses, this time restricting the search volume to the volume defined by the 11,313 voxels showing significant BOLD covariation (voxelwise p≤.01, whole-brain cluster-corrected, p≤.05) with the behavioural measures of multisensory integration. Only the simple visual contrasts resulted in any significant clusters of activation (Figure 5, Bonferroni-corrected across the search volume). Activation peaks were located in the superior and inferior divisions of the lateral occipital cortex, the fusiform, and lingual gyri.

## Discussion

The experiments reported here were designed to determine whether holding and using a tool on one side of space is accompanied by a significant modulation of visual processing for stimuli presented on that side, near to the functional part of the tool. The data indeed supported this conclusion. Clusters of voxels in occipital cortex contralateral to the visual stimuli showed increased BOLD responses when the tip of the tool was held and used next to the stimulus (as compared to when it was used on the side of fixation opposite to the visual stimulus). In a complementary manner, clusters of voxels in occipital cortex ipsilateral to the visual stimuli showed decreased BOLD responses when the tool tip was used next to it, as compared to when used on the contralateral side. Several clusters, in particular in the right hemisphere lingual gyrus, shoed both increased and decreased responses as a function of tool position.

We also identified regions in which the BOLD response covaried significantly with performance on the behavioural multisensory discrimination task. This analysis revealed a number of areas in the medial and lateral superior frontal gyrus, occipital cortex, and cerebellum. Specifically, BOLD response in these areas increased or decreased in linear proportion to the magnitude of multisensory interactions across participants. The response in these areas may reflect several multisensory processes, including the integration of visual and vibrotactile stimuli at the single cell or population level, the detection and resolution of multisensory response conflicts, the generation of responses based upon these conflicting multisensory cues, and any differences in error-related processing across participants.

Finally, we repeated our original analyses in order to examine which of the three experimental variables (visual distractor position, tool tip position, and hand used), or their interactions, most likely contributed to the generation of the significant multisensory integration effects. These final analyses revealed that the position of the visual distractor relative to the visual midline was clearly the most relevant factor for influencing the BOLD response specifically in those brain regions whose response was most tightly coupled with the between-participant behavioural variation in multisensory integration.

### New findings

We instructed the healthy participants in our experiments explicitly to ignore the visual distractors, and to pay attention only, and to respond only, to the type of vibrotactile target presented at the tip of the tool, and felt by the hand holding the tool. The optimal strategy for correct task performance might therefore have been to close one's eyes and to pay attention to the vibrotactile stimuli perceived by the hand holding the tool. However, we instructed participants to keep their eyes open and fixated on a central visual stimulus, monitoring the fixation cross for brief, subtle, and unpredictable changes in luminance, and withholding the subsequent motor response when this occurred.

The participants performed the fixation task with >80% success (chance performance would have been ∼8%, see Figure S3), and showed both significant behavioural effects of multisensory congruency, and BOLD response differences as a function of the relative location of the visual distractors with respect to the functional tip of the tool. These results occurred despite our instructions to the participants to ignore the visual distractors and to respond only to the vibrotactile targets. In summary, this new finding allows us to conclude that using a tool to perceive vibrotactile stimuli has, as one consequence, a shift in participants' spatial attention to the location where the functional part of the tool is held and used, and a subsequent increased BOLD response for visual distractors presented at that location, and a complementary decreased BOLD response for visual distractors presented in the ipsilateral visual field.

These findings using fMRI complement our recent purely behavioural findings reported elsewhere [7]–[9]. In these studies, we showed that holding and repeatedly using a variety of tools results in the preferential processing of visual distractors primarily near the functional parts (typically the distal tips) of the actively used tools. Until the present fMRI data were collected, it was difficult to discern, *a priori*, whether such behavioural results were due predominantly to shifts in visuospatial or multisensory attention to the position where the functional part of the tool were held, or due, at least in part, to activation of hand-centred or tool-centred multisensory representations, for example in posterior parietal or premotor cortices [10], [44], [45]. The fMRI data presented here clearly support the former possibility – that the reported behavioural effects of tool use may be due predominantly to relatively low-level attentional effects of the position of a tool on the processing of incoming sensory stimuli in occipital cortex.

The second major new finding in the present report is that the multisensory interactions observed in a previously well-studied behavioural congruency task [9], [18]–[20] are strongly and significantly related, across participants, to neural processing in the right medial and superior frontal cortex, the midline and lateral cerebellum, and bilateral occipital cortex. Different nodes in this network are very likely to be involved in the detection and resolution of the multisensory response conflicts, in response selection, and in error-related processing that arises in such congruency tasks (e.g., particularly in the medial frontal cortex [35]). However, the BOLD responses in other regions within this network, particularly the lateral occipital cortex, were also shown to depend significantly on the location of the visual distractor stimuli. We take this as evidence that visual or multisensory processing in these areas is tightly linked to the generation of the behavioural effects of multisensory congruency between the visual and vibrotactile stimuli.

### Possible criticisms of the present study

A number of possible methodological and theoretical criticisms of the present work need to be addressed explicitly. These criticisms have been raised by various reviewers and commentators on our work in the past, and, although we have dealt with similar criticisms elsewhere [7]–[9], we provide a brief discussion of these issues here.

#### What is tool use?

This simple-sounding question is in fact extraordinarily difficult to answer. The interpretation of the present results and their relevance to other studies of ‘tool use’ clearly requires an answer to this question. Benjamin Beck is a renowned authority on the use of tools in the non-human animal kingdom, and in his 1980 book [46] on the topic he concluded: “After 15 years of trying [to provide a definition of tool use], I'm unhappy to report that I have not been totally successful.” [46, p. 4]. We concur with the thoughtful conclusions and the caveats raised by Beck, and believe that attempts to define tool use simply, non-arbitrarily, and non-circularly are fraught with difficulties. One option could be, for example, to resort to a dictionary definition, with the Oxford English Dictionary (OED) offering: “any instrument of manual operation”, where instrument is defined as an ”object used for a given purpose”. But this definition seems, to us at least, somewhat vague to be useful in a scientific context. A computer keyboard, for example, is a manually-operated object used for the purpose of data entry, but the sensory-motor interactions required in the prototypical use of a keyboard are rather different from those required to use a screwdriver, a rake, a pointer, or the canes used to navigate by the blind. According to the OED definition, the participants in our study were clearly manually operating an instrument for a specific purpose – without holding, correctly orienting, exerting downwards pressure, and moving the stick at the correct times, the participants in our task could not have performed correctly. From the fact that they did indeed perform the tasks well above chance, we can conclude that tool use was occurring during the fMRI sessions.

As compared to dictionary definitions, in the academic community studying the effects of a variety of tool use tasks on sensory-motor and multisensory interactions, tool use has rarely been defined explicitly. We have recently provided [47] one possible definition, extrapolating from Beck's definition in order to apply it both to humans in general, and to the scientific literature on tool use and peripersonal space in particular. In that definition, we aimed to draw a line (albeit an arbitrary one) between a number of tasks that either did or did not constitute tool use. Such a definition can of course never be the final word, particularly given the rate at which technology develops, especially that of tele-surgical and brain-computer interface devices. We believe, however, that our definition of tool use is both sufficiently liberal and sufficiently conservative to allow clear hypothesis-driven questions concerning multisensory attention (and multisensory peripersonal space) to be answered in a pragmatic way. Furthermore, we are extremely wary of the possibility that one might choose to define tools rather flexibly, on the basis of the *results* of particular studies of tool use – i.e., whether the results are consistent or inconsistent with one's beliefs about the effects of tool use on, for example, representations of peripersonal space or on multisensory attentional processes [48]. Since the key researchers working in the scientific field addressed by the present study must certainly agree that the task we used clearly constituted tool use (e.g., compare the wide variety of tool use tasks reported by such researchers [3]–[17]), we feel that this question over definitions is tangential to the relevance and importance of the present findings. In any case, if one were to decide that our task was not really ‘tool use’, we have still succeeded in showing that the position, relative to visual distractors, of the functional part of a manually-held object used to perceive distant vibrotactile stimuli significantly modulates the BOLD response in the occipital cortices, and that these modulations are most likely due to spatial shifts of attention towards the functional tip of that object. We see no reason why other, genuine, or more complex, forms of tool use would not also involve such shifts of attention to the functional part of the tool. This possibility, however, needs to be assessed in future research.

Finally, concerning definitions of tool use, we note the recent paper by St. Amant & Horton [49]. In their detailed theoretical discussion of how to define tool use in the animal and human behavioural literature, St. Amant & Horton also revised Beck's now classic definition of tool use to include those instances of tool use which “mediate the flow of information” between the tool user and the environment, including both direct physical interactions with objects, and communicative gestures. This ‘mediation of information flow’ through direct physical interactions with objects fits very well with the working definition of tool use that we proposed [47].

#### Visual attention at the tip of the tool, or a non-linear supra-additive interaction between multiple visual stimuli?

One of the reviewers of this manuscript pointed out that the increased BOLD responses in (contralateral) occipital cortex when the tool was held and used next to the visual stimulus could be due either, as we suggested, to the effects of spatial attention to the tip of the tool (and thus to the visual distractor presented at the same location), or else to an interaction between two separate visual stimuli (the visual distractor and the tip of the tool respectively), arising, simply and trivially, from the presence of more visual stimulation in that portion of the visual field. The interaction between the two visual stimuli could arise in one or both of two ways:

1) If the BOLD response in occipital cortex summed up separate, independent visual inputs in a non-linear and supra-additive fashion (the effects would have to be supra-additive because: a) The tip of the tool was present in the visual field for 12 or 36 s before the visual distractors were illuminated, likely giving sufficient time for the BOLD response to habituate to any simple visual effect of the tip of the tool alone, and; b) The critical contrasts were performed between conditions with identical visual distractors on the same side of space);

2) If the visual distractor stimulus increased the illumination of the tip of the tool relative to its background, thus increasing the overall illumination present in that portion of space. We can make four arguments against these possibilities.

First, regarding non-linear summation. Probably due to the habituation and saturation of the BOLD response, the large size of functional imaging voxels, and the presence of heterogeneous cell populations within a given voxel, studies of *multisensory* interactions in neuroimaging very rarely find any evidence for supra-additive summation, despite numerous clear examples of multisensory supra-additivity in single and multi-unit recordings, local field potentials, and in behavioural multisensory interactions [27], [33]. More commonly, additive or sub-additive effects are found in the BOLD response to independent stimuli, even when those stimuli are congruent with each other and presented close to each other in space and time. Similarly, and more directly relevant to the present concern, in one detailed and intensive study of the summation of responses to separate visual stimuli within a portion of retinotopic primary visual cortex, the BOLD response was found to sum *linearly* in two human subjects [50]. Given these findings, and assuming that no simple or physical visual interaction effects occurred (see the next paragraph), the possibility of non-linear and supra-additive spatial summation of separate visual inputs can be disregarded.

Second, it is possible that the visual distractors illuminated the tip of the tool, and that this increased illumination on the same side as the visual distractor resulted in additional BOLD response in contralateral occipital cortex. Note that such an explanation would not require any non-linear or supra-additive summation of BOLD responses, so is independent from the first possibility discussed above. Our experiments were performed in a dimly-lit scanner room. We did not make any measurement of the total illumination present at different positions in the visual field during the eight experimental conditions studied. We can only note here that the visual distractor stimuli were positioned at the rear of an unpainted aluminium box, mounted on a transparent acrylic table over the participant's legs. Both of these pieces of apparatus would have reflected some of the light of the distractor into the scanner bore. During the experimental set-up, we asked participants whilst inside the scanner bore to inform us if the visual distractor stimuli were being reflected off either surface, and if they were, we adjusted the distractor positions to remove these secondary visual inputs. By contrast with the smooth, reflective surfaces of the aluminium box and the acrylic table, the tip of the tool was wooden, rough, and less reflective. Thus, it is unlikely that any simple visual effects of reflection or illumination could explain the modulations of BOLD response in occipital cortex, since the less-reflective surface of the distal tip of the tool was *occluding* the more-reflective surfaces of the aluminium and acrylic apparatus when the tip of the tool was on the same side as the distractor.

A third point to note is that we observed both *contralateral increases* in BOLD response as a function of tool position (i.e., a tool used on the left side *increased* the BOLD response in *right occipital cortex* to left visual distractors as compared with when the tool was used on the right), and *ipsilateral decreases* in activation (i.e., a tool used on the left side *decreased* the BOLD response in *left occipital cortex* to left visual distractors as compared with when the tool was used on the right side). In order to explain our findings, any simple effects of two visual stimuli would therefore have to show both non-linear supra-additive *positive* BOLD in contralateral occipital cortex, and non-linear supra-additive *negative* BOLD in ipsilateral occipital cortex. This double and hemispherically-symmetrical non-linearity in the BOLD response is thus doubly unlikely as an explanation for our reported effects. Furthermore, in the presence of an attentionally-demanding RSVP task performed at central fixation, the BOLD responses elicited by large, high-contrast peripheral visual distractors in areas V1 to V4 in one hemisphere were unaffected by the presence of *ipsilateral* visual distractors, suggesting that ‘surround suppression’ does not operate inter-hemispherically [22]. This implies that the deactivations that we observed cannot be due to the mere presence of visual stimulation in the ipsilateral hemispace, but rather require an attentional explanation [21].

Fourth and finally, as detailed in the Results section, the regions of cortex in which the BOLD response was modulated significantly as a function of tool tip position, concur well with those activated in previous studies of visual or multisensory spatial attention, which is known to operate in a ‘push-pull’ manner, with both increases and decreases of attention-related activation in extrastriate cortex [21].

To conclude, we note that additional experiments in which participants performed both a passive and an active tool use task, under similar experimental conditions as reported here would be required in order definitively and finally to rule out the possibility of bilateral non-linear supra-additive spatial interactions due simply to the visual presence of the tip of the tool. However, given: 1) The generally linear summation of BOLD responses; 2) The fact that the tip of the tool was not highly visible and was in fact less reflective than the background; 3) The presence of both contralateral increases and ipsilateral decreases in BOLD response as a function of tool position, and; 4) The locations of the reported BOLD modulations relative to other studies of spatial attention, we believe that the attentional explanation is the more parsimonious one, since spatial attention has repeatedly been shown to lead to both increases in BOLD response for stimuli presented at the attended, and decreases in BOLD response for stimuli presented at unattended locations.

#### Absence of significant hand-centred or tool-centred multisensory interactions or BOLD responses

The absence of evidence for a particular process can never, of course, be taken as evidence of the absence of that process. We did not find significant clusters of activation, or any strong indications that hand-centred multisensory processes were operating during our tool use task, under the conditions we studied, despite the presence of strong visual-vibrotactile behavioural interactions, and the indications from previous studies that such interactions may be hand- or tool-specific [9], [10], [12]. This is surprising if one believes that multisensory interactions during tool use occur in hand-centred reference frames, and that such interactions occur in or result from processing in parietal and or premotor cortices [10]. We do not hold this belief. Rather, we believe that many, if not all, of the reported effects of tool use on multisensory integration may in fact be due predominantly to eye-centred mechanisms of multisensory spatial attention, and that the locus of these effects may be in relatively ‘low-level’ or ‘early’ sensory (visual) cortices. We believe that the present data, our previous behavioural results, and many of the published results on the multisensory consequences of tool use from other laboratories, are most clearly and parsimoniously accounted for by such eye-centred effects of multisensory spatial attention, rather than by, for example, hand-centred mechanisms of peripersonal space. However, since the majority of studies in this field have not been able experimentally to distinguish between these alternative hypotheses, future studies are clearly required in order to resolve any apparent contradictions that remain in the literature.

Several other factors may account for the absence of hand-centred effects in the present dataset. First, the design of our study, or the fMRI protocol we used, may have been insufficiently powerful or sensitive to detect the very subtle, sparsely-distributed, or spatially very limited effects that have been proposed to generate the significant and apparently hand-centred multisensory interactions reported elsewhere. This possibility can only be confirmed with positive evidence showing that such processes do indeed exist and are measurable, while also providing evidence to rule out alternative possibilities such as the attentional hypothesis discussed here. Such evidence is not yet available, and we must therefore await future studies. If such effects do exist, yet are very weak or subtle, it raises the questions as to whether they can indeed explain all of the many reported multisensory consequences of tool use behaviours, and why the more powerful and more easily-detected effects of spatial attention are behaviourally and neurally less important or effective in this regard.

Second, it may be possible that the visual stimuli we used (static, flashing LEDs) are simply not able to activate the regions of parietal and premotor cortex that are thought to be involved in mediating sensory processing during or following tool use. This is an important possibility, which needs to be tested in future research. For now, we simply highlight the fact that, in the neuropsychological literature on the effects of tool use on cross-modal extinction, static flashing LEDs and three-dimensional rapid movements of an experimenter's finger have been shown, in one study, to result in comparable levels of visual-tactile interactions [14]. The possible implications from this study are either: a) That the stimuli used (both the rapidly moving fingers and flashing LEDs) were also insufficient to activate parietal and premotor cortex (since static LEDs and moving fingers produced comparable results) and that the reported effects must therefore have depended upon mechanisms of spatial attention and modulation of activity in occipital cortex, or; b) That LEDs are indeed sufficient to activate parietal and premotor cortex in an equivalent manner to rapidly moving fingers. The results of our study support the former, attentional interpretation.

Finally, it is possible that the specific kinds of tool used, or the specific tool use task performed, have a direct bearing on the kinds of multisensory interactions that occur, and the neural processes that are involved. This is almost certainly true in some respects, for example, relating to the well-known differences in the neural control of precision vs. power grasping movements, or between the reaching/transport and grasping/hand-shaping components of target-directed movements, which undoubtedly will differ between different tools. However, we only wish to note here that, with regard to the published evidence concerning the effects of a variety of tool use tasks on sensory-motor and multisensory processes, Maravita and Iriki [10] concluded:

“Intriguingly, whilst in some studies on humans the reported behavioural effects of tool-use occurred without any specific training … in other studies substantial tool-use training was required to elicit these effects … It might be that simple acts, like pointing or reaching with a stick will show behavioural effects without training, whereas more complex tasks involving dexterous use of a tool, such as retrieving objects with a rake require some training before any behavioural effects will emerge.” [10, p. 84].

We agree in general with these sentiments, however we remain cautious about the possibility that successful tool use is being defined here based upon the results (the ‘behavioural effects’) observed in the reported experimental settings, rather than upon the tool used and/or the tool use task being performed. The question therefore arises: Given a particular or novel tool use task, such as the one used in the present report, for how long should one continue the tool use training in order to test the hypothesis, for example, that tool use changes multisensory processing? If the answer is either: a) ‘Until multisensory processing changes’ or; b) ‘Until the well-known, prototypical effects of tool use emerge’, then it seems, at least to us, that such a hypothesis would be impossible to refute.

### Conclusions

Our results have clear and important implications for how multisensory stimuli are processed during and following the use of simple hand-held objects as tools. The present results suggest that tool use is associated with an automatic shift of spatial attention to the location where the functional part of the tool is used. The position of the functional part of the tool relative to visual distractor stimuli modulated the BOLD response, both positively and negatively, in portions of visual cortex likely comprising retinotopic areas V1–V4 and VP, and most prominently in the right hemisphere lingual gyrus, which also showed a significant between-participants covariation between the BOLD response and the behavioural measures of multisensory integration. This spatial attentional shift occurred despite the fact that participants were specifically instructed to ignore the visual distractor stimuli, and to attend to and respond only according to the vibrotactile stimuli. The consequence of this shift of spatial attention was that activity in occipital cortex was modulated in a manner consistent with previous studies of the *voluntary* orienting of spatial attention, enhancing activity contralateral, and suppressing activity ipsilateral to the visual distractors. This modulation of activity in retinotopic portions of occipital cortex may represent the early selection of relevant and suppression of irrelevant visual stimuli for the control of tool use actions.

## Materials and Methods

### Participants

Twenty-one participants were recruited and paid twenty pounds (UK Sterling) per scan for their participation. All reported being right-handed, having normal or corrected vision, normal tactile sensation, and no neurological or psychiatric abnormalities. Fourteen participated in Experiment 1 (right-hand tool use, 5 female, aged 21–36 years, mean±s.e.m. = 24.9±1.1 years, mean±s.e.m. handedness laterality quotient (LQ) = 76.4±6.9, [51]), and thirteen participated in Experiment 2 (left-hand tool use, 5 female, aged 20–30 years, mean±s.e.m. = 25.8±0.8 years, mean±s.e.m. LQ = 70.6±7.0, including six participants from Experiment 1). All experimental procedures were approved by the local National Health Service ethics board and were conducted in accordance with the Declaration of Helsinki. All participants gave informed written consent to participate and were screened for MRI-safety criteria before being scanned. Behavioural data from one participant in Experiment 1 were lost due to human error.

### Apparatus & materials

#### Piezoelectric vibrotactile stimulators

Two custom-built MR-compatible piezoelectric-ceramic vibrotactile stimulators driven by a custom-built waveform generator were used to deliver vibrotactile stimuli. Each consisted of an aluminium box (5.5×2.2×8.0 cm) containing a 2 cm piezoelectric-ceramic element, vertically displacing a plastic rod ∼1 mm. The vibrating surface of the stimulus was 19.6 mm^2^. Stimuli were presented at ∼200 Hz [52]–[53]. A small rubber semi-circular ‘guide’ was positioned on top of the vibrotactile stimulus to facilitate tool positioning during the experiment.

#### Additional apparatus

The vibrotactile stimulator boxes were attached by Velcro™, 15 cm either side of the middle of an acrylic table (15×75×45 cm). One red LED (8 mm diameter, 660 nm, 550 mcd, 60° viewing angle) was positioned 1 cm above the vibrotactile stimulator on each side. The tool was a cylindrical wooden dowel (8 mm diameter×750 mm length). A rear-projection screen was positioned over the legs of the participants, ∼1.5 m from their eyes (Figure 1E). Responses were collected with a MRI-compatible button box, held by the participant in the hand opposite to the one holding the tool. The stimuli were controlled and the responses were collected using Presentation software (Neurobehavioural Systems Inc., Albany, USA). The experimental apparatus caused no detectable artefacts in the fMRI data.

### Design

Each participant performed two experimental runs per hand tested. Each run consisted of 16×24 s ‘task’ blocks, interleaved with 16×18 s ‘rest’ blocks. Each run lasted 681 s, including an initial 9 s for scanner equilibration. Each block consisted of one of four visual-vibrotactile conditions, resulting from the factorial combination of the two condition variables: 1) Tool tip and vibrotactile target position (TL: left, TR: right), and; 2) Visual distractor position (VL: left, VR: right). The four conditions were as follows: 1) TLVL; 2) TLVR; 3) TRVL; 4) TRVR (Figure 1). They were run in separate blocks of trials, in ascending order (1-2-3-4-1-2…) in one run and in descending order (4-3-2-1-4-3…) in the second run, with the sequence of four blocks repeated four times per run. This fixed block order was used to keep the number and timing of tool movements constant within and between participants. The four possible starting conditions and two possible starting sequence orders were fully counterbalanced across participants. In post-hoc analyses, peak voxels within significant clusters of activation, in which the BOLD response showed significant main effects of block order, or interactions between block order and the experimental conditions of interest, were identified, and are not discussed here due to possible artefactual or theoretically uninteresting effects. Each block contained four trial types, resulting from the factorial combination of the two trial variables: 1) Vibrotactile target stimulus type (continuous vs. pulsed stimuli), and; 2) Visual distractor stimulus type (continuous vs. pulsed). Continuous stimuli were 200 ms in duration. Pulsed stimuli were also 200 ms in duration, but contained a 70 ms gap with no stimulus in the middle (i.e., 65 ms ON, 70 ms OFF, 65 ms ON). Each of the four trial types was presented three times per block of trials, in a pseudorandomised order, with one multisensory stimulus presented every 2 s. Within each block, one additional trial (a ‘fixation’ trial, see below) was pseudorandomly interleaved within the sequence of twelve trials.

The right hand tool use experiment was run first. The left hand tool use experiment was run several weeks or months later. Participants were either tested on both experiments, or matched for age, sex, and handedness score between experiments (between-experiments t-tests, t(12)<1, ns). We had no *a priori* hypotheses concerning differences between the effects of using left and right hands, and assumed equal variances for the two conditions, allowing us to perform paired analyses.

### Procedure

The participants lay supine in the scanner, viewing the experimental apparatus and screen through a mirror mounted on the head coil (Figure 1E). The participants' arms, head, and legs, and the stimulus table were supported with soft padding in order to minimise movement and the spread of vibration. The visual and vibrotactile stimulators were adjusted, immediately prior to the scan, to lie at the distal tip of the tool held in the participants' hand, and were positioned in order to minimize shoulder, arm, and hand movements during the experiment. The distance of the stimuli from the participants' eyes therefore depended on the length of their arms. The maximum difference in distances between the left and right stimuli and the participants' eyes was ∼6 cm: The left stimuli were closer to the participant for right hand tool use, and the right stimuli were closer for left hand tool use. These differences were nevertheless constant across the four experimental conditions, and the crucial comparisons of interest (the effect of tool side within the cluster defined by the main effect of visual distractor side) were performed separately for left and right visual distractor positions, thus fully balancing any simple effects of visual distractor distance or other possible, but irrelevant, differences between left and right visual distractors (e.g., visual angular size or luminance).

#### Task blocks

In the task blocks, participants performed a vibrotactile discrimination task and a visual fixation-monitoring task concurrently.

#### Vibrotactile discrimination task

The participants were instructed to hold the distal tip of the tool in contact with the active vibrotactile stimulator on one side (left or right) throughout a block of trials, while maintaining fixation on a white cross (3×3 cm) presented centrally at the bottom of the rear-projection screen. Participants were instructed to respond as quickly and as accurately as possible to the vibrotactile target stimuli by pressing one of the two buttons: The left button for continuous, and the right button for pulsed stimuli, while maintaining central fixation and trying to ignore the visual distractor stimuli. The visual distractor stimuli were irrelevant to the task and were non-predictive of the vibrotactile target type.

#### Fixation-monitoring task

The visual fixation cross dimmed for 250 ms, from white (100% screen brightness) to grey (70%) once during each task block. Following a fixation dimming event, the participants were required to withhold their response on the subsequent trial in the current block.

#### Rest blocks

At the beginning of each rest block, the fixation cross was replaced by a white chevron (< or >) indicating the position (left or right, respectively) of the active vibrotactile target stimulator for the next task block. For half of the blocks, this required no change of the position of the tip of the tool, and for the other half, the participants were required to move the tip of the tool to the side indicated by the chevron as quickly as possible, while making the minimum of body movements (i.e., by moving only their wrist and fingers). After 5 s, the chevron was replaced with the fixation cross, which remained in place for a further 11 s, was extinguished for 500 ms, then re-illuminated for the last 1500 ms of the rest block as a cue for the participant to prepare for the impending task block. The participants were instructed to remain as still as possible, and to maintain visual fixation on the fixation cross throughout the rest block. It is very important to note here that the tool tip was in position and remained static for at least 12 s (and for half of the blocks, 36 s) before each and every block of experimental trials. This ensured that any changes in BOLD activity recorded in the task blocks relative to the baseline ‘rest’ blocks could not simply be due to the position of the tool or to the tip of the tool acting as an additional ‘visual’ stimulus (see also the Discussion). Task-related changes in BOLD response could therefore only be due to the main effects of visual distractor position, vibrotactile target/tool use location, the interaction between these variables, or to a main effect of task performance, which is of little theoretical interest and not reported here.

One or two days before the scanning session, participants trained on the tool use and fixation monitoring tasks for 10–20 minutes in a simulated scanner environment, lying supine, holding the same tool and discriminating the same target vibrations from the same apparatus. Recorded scanner noise was played in the background or over headphones. Such periods of training on tool use tasks has often been argued to result in significant changes in multisensory integration in peripersonal space [10].

### Magnetic resonance imaging

Images were acquired on a Siemens Sonata 1.5T magnet. Echoplanar (EPI) T2*-weighted functional images were acquired with the following parameters: Repetition time (TR) = 3 s, echo time (TE) = 50 ms, voxel size = 3×3×3 mm, 35 contiguous axial slices acquired dorsally to ventrally, matrix size = 64×64. Functional data acquisition and the behavioural task began 9 s (three TRs) after the onset of the run. 224 whole-brain volumes were acquired per run. High resolution, T1-weighted structural images were acquired with the following parameters: TR = 12 ms, TE = 4.76 ms, voxel size = 1×1×1 mm.

### Analysis

#### Pre-processing

All fMRI analyses were performed with FEAT (fMRI Expert Analysis Tool) Versions 5.63 or later, part of FSL (FMRIB's Software Library, http://www.fmrib.ox.ac.uk/fsl). The following analysis was applied to each functional run; Slice-timing correction using Fourier-space time-series phase-shifting; Motion correction using MCFLIRT [54]; Non-brain removal using BET [55]; 3D spatial smoothing using an isotropic Gaussian kernel of 8 mm full width at half maximum (FWHM); Global (volumetric) multiplicative mean intensity renormalization; High-pass temporal filtering (Gaussian-weighted LSF straight line fitting, with sigma = 54 s, corresponding to a low-pass cut-off of 1/108 Hz). Time-series statistical analysis was carried out using FILM with local autocorrelation correction [56]. Registration of each participant's functional T2*-weighted to high resolution T1-weighted scans and subsequently to the MNI52 standard brain template was carried out using FLIRT, with 7, and 12 degree-of-freedom linear transforms, respectively [54], [57].

#### Within-participant analysis

The two time-series of functional data for each participant were modelled with a boxcar block design (24 s ON, 18 s OFF) convolved with a canonical (double-gamma) haemodynamic response function, and delayed by 6 s. Four regressors of interest corresponding to the four conditions (TLVL, TLVR, TRVL, & TRVR), and seven regressors of no interest (the temporal derivatives of the four main regressors, plus leftward tool movements, rightward tool movements, and fixation dimming events) were included in the model for each functional run. The two sets of contrast images obtained from each run for each participant were submitted to a higher analysis using a fixed effects model, by forcing the random effects variance to zero in FLAME [58]–[59]. The results of this within-participant analysis were passed-up to a higher-level between-participants (group) analysis.

#### Between-participants analysis

The final group analysis was carried out using mixed effects (in which participant was a random effect) FLAME, in MNI152 template space, with final voxel dimensions of 2×2×2 mm. Statistically significant responses were determined by applying an initial Z-value (i.e., Gaussianised-t) cut-off as a cluster creation threshold, and assessing the size of contiguous clusters of voxels against Gaussian random field theory, correcting for multiple comparisons across the search volume to p≤.05, corrected. Several analyses also used Bonferroni corrections for multiple comparisons, as detailed in the Results section. Three different analyses were performed to assess visual, tool-dependent, and multisensory effects of interest.

In the first analysis, data from all eight experimental conditions (i.e., the four main conditions, for both the left and the right hand tool use experiments) were entered into a 3-way analysis of variance (ANOVA) with the variables hand (left, right), tool side (left, right), and visual distractor side (left, right). Weighted contrasts for all effects of interest were assessed with an initial Z-value threshold of Z = 2.33, p≤.01, and whole brain cluster corrected to p≤.05. The following contrasts were performed (Figure 1B–D): 1) The simple effects of visual distractor position were tested with two contrasts, one for the left [(TLVL+TRVL)>(TLVR+TRVR)], and one for the right visual distractors [(TLVR+TRVR)>(TLVL+TRVL)] (Figure 1B); 2) The effects of tool position were tested with two contrasts, restricted to the regions within the clusters defined by the simple effects of visual distractor position: [TLVL>TRVL], and [TRVR>TLVR]. The simple main effects of tool position (Figure 1C) were also tested; 3) Finally, the presence of hand-centred or tool tip-centred activations was assessed with the interaction between visual distractor position and tool tip position (Figure 1D). This was performed both separately for each experiment (i.e., for each hand used), collapsing across the two experiments, and with experiment as an additional variable in a three-way analysis.

In the second analysis, the mean of each of the eight experimental conditions was modelled out as a regressor of no interest, and two sets of eight behavioural measures (crossmodal congruency effect derived from RTs and percentage errors, with the across-participant mean subtracted per condition) were entered as predictors in separate analyses. Areas with BOLD responses that covaried significantly (either positively or negatively) with the behavioural measures of multisensory integration, were assessed across the whole brain, separately for RT and error score predictors.

In a third analysis, the first set of analyses (i.e., the contrasts involving the experimental variables of visual distractor position, tool position, and hand used) were repeated, but restricting the search volume to those regions in which the BOLD response varied significantly as a function of the behavioural measures of multisensory integration (i.e., the inclusive sum of significant clusters with either positive and negative covariation between BOLD response and RT or error measures of multisensory integration).

For all analyses, significant activation peaks of interest in the group statistical maps were interrogated using FEATQUERY: A mask was created manually in MNI template standard space, incorporating several voxels surrounding the peak voxel in order to create a mask, typically of 5–20 voxels (0.14–0.54 cm^3^) in volume after transformation back to each participant's native space. The number of voxels in the mask was determined by decreasing the Z-statistic threshold of the group activation map until at least five contiguous voxels around the peak were above threshold (this process was performed solely to ensure that sufficient voxels remained after transformation of the mask from MNI template standard space sampled at 2×2×2 mm, to the participants' native space sampled at 3×3×3 mm). The percentage signal change was averaged over those voxels for each of the eight experimental conditions separately, and further analysed with four-way repeated measures ANOVA (including the additional variable, task sequence order) in order simply to confirm the directions of particular effects following the whole-brain analyses. Since these analyses of signal change were biased by the prior selection of peak voxels in a given contrast, these exploratory signal change analyses were used predominantly: a) To *exclude* groups of voxels from subsequent interpretation which showed artefactual effects (for example, those peak voxels lying near the edges of the brain or ventricles); b) To exclude groups of voxels from subsequent interpretation which showed any significant effects or interactions with the order in which the tasks were performed (p≤.01), and; c) To assess whether significant contrasts resulted from activations, deactivations, or both directions of modulation relative to the baseline. Data from peak voxels in which artefactual effects of, or interactions with, block order were not included or discussed in the analysis or report. This process was performed after the main statistical analyses reported in the text. No other significant clusters were observed.

Two additional *post-hoc* analyses were performed based on the raw timeseries data of a cluster of ‘seed’ voxels in the right hemisphere lingual gyrus, centred on the MNI coordinate (20, −68, −2). The first analysis assessed the ‘functional connectivity’ between signal in this area and other brain areas by searching for voxels whose signal covaried with signal in the seed voxels. The second analysis assessed the ‘effective connectivity’ as a function of the task versus rest: The raw timeseries data were multiplied by +1 for signal reflecting task periods (i.e., delayed by 6 s), and −1 for signal related to rest periods. In each case, the demeaned timeseries was entered as a regressor in a first-level analysis without temporal filtering, convolution with the double-gamma HRF, or including temporal derivative regressors. Higher-level analyses were performed as described above.

All voxel coordinates in the text and figures refer to the MNI152 standard brain template in MNI152 template space.

## Supporting Information

Text S1 (0.05 MB DOC)

Figure S1 Behavioural data. Data show the mean±s.e.m. magnitude of multisensory integration effects (MSI, defined as performance on incongruent - congruent trials), across 13 participants per experiment (hand). Filled grey columns: visual distractor on the left of fixation. Open columns: visual distractor on the right of fixation. Left half of each panel: tool held in the left hand. Right half: tool held in the right hand. Left half of each of these sub-panels: tool tip positioned on the left of fixation. Right half: tool tip positioned on the right of fixation. A. RT. B. Errors.
(0.16 MB TIF)

Figure S2 Activity negatively correlated with multisensory integration (% error measures). Clusters of activation show predominantly right hemisphere brain areas in which the BOLD response significantly negatively covaried with the magnitude of multisensory integration across participants, overlaid on a standard MNI template brain. Voxels were thresholded at ≥2.33, p≤.01, and the resultant clusters were corrected for spatial extent across the whole brain, p≤.05. For display purposes the threshold was increased to Z≥3.09, p≤.001. The data panels show percentage signal change against baseline (y-axis) against the magnitude of multisensory integration derived from percentage error measurements. For display purposes, data were pooled for the left hand (blue circles) and the right hand (red triangles). MSI: multisensory integration.
(1.46 MB TIF)

Figure S3 Evidence that the participants maintained central visual fixation for the majority of the time during the experimental procedures. A. Percentage correct fixation task performance for the data of 13 participants in each experiment (Left hand tool use, Right hand tool use, along the x-axis). During the blocks of experimental trials, participants were required to monitor the fixation cross for brief (250 ms) decreases in brightness. In response, participants were instructed to omit their response to the target on the subsequent trial. The broken horizontal line indicates chance performance at 8.33% correct. B. Simple effects of visual distractor side (VL>VR: left visual distractor>right visual distractor, cool colours; VR>VL: right visual distractor>left visual distractor, hot colours), for each participant in each experiment (L1–13: left hand tool use; R1–14: right hand tool use). Z-statistic contrast images were thresholded (Z≥2.33, p≤.01, uncorrected), and overlaid on each participant's anatomical scan in their native space. One slice (selected from the approximate MNI Z-coordinates +8 to +20) is shown for each participant, illustrating clusters of activation in occipital cortex.
(2.65 MB TIF)

Table S1 (0.05 MB DOC)

Table S2 (0.05 MB DOC)

Table S3 (0.06 MB DOC)

Table S4 (0.05 MB DOC)
