# Supplementary material for: The Multisensory Attentional Consequences of Tool Use: A Functional Magnetic Resonance Imaging Study
Source: PLoS One. 2008 Oct 29;3(10):e3502. doi: 10.1371/journal.pone.0003502 (PMC2567039; doi:10.1371/journal.pone.0003502)
Supplement: Text S1 — (0.05 MB DOC) [file pone.0003502.s001.doc]

**Nicholas P. Holmes, Charles Spence, Peter C. Hansen, Clare E. Mackay, Gemma A. Calvert**

**The Multisensory Attentional Consequences of Tool Use: A Functional Magnetic Resonance Imaging Study**

#### SUPPLEMENTARY MATERIALS

**Supplementary Results**

##### Behaviour

*Catch trials and missed targets*. For thirteen participants in the right hand tool use experiment, the mean±s.e.m. number of missed catch (fixation) trials out of 32 was 7.2±2.2 (22.5±6.9%), and the number of missed targets out of 384 total targets was 6.3±2.1 (1.6±0.6%). For thirteen participants in the left hand tool use experiment, participants missed 4.4±1.1 (13.8±3.4%) fixation trials, and 22.5±6.5 (5.9±1.7%) targets. Chance performance on the fixation task would have resulted in 91.7% misses. Significantly more targets were missed in the left than right hand tool use experiment (2-tailed paired t-test, t(12)=2.53, p=.028), but there were no other significant differences between experiments.

*Reaction times and errors*. The data from both experiments (twelve complete data sets per experiment, matched for sex, age, and handedness (all between-experiment ps>.10)) were entered into a single multivariate analysis of variance (MANOVA), with the variables hand (left, right), tool position (left or right), target type (single, double), visual distractor side (same or different), and visual distractor congruency (congruent, incongruent). There were significant multivariate main effects of target type, F(2,10)=4.47, p=.041, and congruency, F(2,10)=21.03, p=.0003, and interactions between tool position and congruency, F(2,10)=6.87, p=.013, and between target type and congruency, F(2,10)=13.25, p=.002. No other effects or interactions were significant (Supplementary Figure S1).

Follow-up ANOVAS performed on the univariate data revealed no significant effects of target type either for RT, F(1,11)<1, (mean ± s.e.m. single=742±31ms, double=748±2ms), or error data, F(1,11)=2.99, p=.111 (single=10.6±3.0%, double=5.3±1.1%) when considered separately. The effect of target-distractor congruency was significant for both RTs, F(1,11)=45.29, p=.00003 (congruent=706±26ms, incongruent=784±32ms), and for errors, F(1,11)=14.06, p=.003 (congruent=1.9±0.3%, incongruent=14.0±3.2%), with worse performance on incongruent than congruent trials. The interaction between tool position and congruency approached significance both for RTs, F(1,11)=3.52, p=.088, and for errors, F(1,11)=4.59, p=.055. For both measures, the difference between incongruent and congruent trials was smaller when the tool was held on the left (mean ± s.e.m. RT differences=70±11ms, error differences=10.9±0.4%) as compared to on the right side (RT differences=86±14ms, error differences=13.4±0.3%). Finally, the interaction between target type and congruency was significant for RTs, F(1,11)=25.49, p=.0004, and for errors, F(1,11)=4.83, p=.050. In both cases, congruency effects were larger for single vibrotactile targets (mean ± s.e.m. RT differences=112±16ms, error differences=18.1±0.6%) than for double targets (RT differences=44±10ms, error differences=6.1±0.2%) (cf. ref [S1]).

##### BOLD responses

*Negative covariation between behavioural multisensory integration and BOLD activation.* A contrast was performed to examine brain regions showing a negative covariation with multisensory integration expressed as percentage errors. This contrast revealed two clusters of activation, one in the right inferior parietal lobule supramarginal gyrus (Z=-5.60, BA39/40, [56, -50, 38]), and the other cluster bilaterally covering portions of the posterior cingulate cortex, precuneus, cuneus, and the parietal-occipital sulcus (Supplementary Figure S2). In general, these clusters contained voxels showing an increasingly negative BOLD response (with respect to the baseline fixation condition) as the magnitude of the multisensory integration effect increased across participants. These deactivations (with respect to rest) may reflect one or more of several processes. First, decreased activation in these areas may reflect a lower ability to attend selectively to touch and to ignore visual information, resulting in larger visual-tactile congruency effects. Second, decreased activation may reflect increased task difficulty in general, in areas associated with resting state or ‘default’ networks [S2-3]. Participants showing the greatest deactivations in these areas had the largest behavioural deficits in RT and error scores for incongruent as compared to congruent stimuli – these participants did worse overall, and, by inference, found the task most difficult.

##### Eye movements and eye position during the experiments

We did not measure eye movements during the fMRI recording sessions, however we are confident that eye position was maintained on the central fixation cross for the great majority of the time and for all the participants studied. First, all participants were required to perform an additional fixation monitoring task during every block – after the fixation cross dimmed from 100% to 70% brightness, the participants were required to withhold their response to the next vibrotactile target stimulus. In the first experiment (right hand tool use), participants withheld their responses correctly on a mean of 77.5±6.9% of trials. In the second experiment (left hand tool use), correct withholding occurred on 86.3±3.4% of trials (Supplementary Figure S3A). Chance performance was 1/12 = 8.33% correct. The higher error rate for the fixation task in the right hand tool use experiment was due to a single participant who misunderstood the instructions and withheld the response to the second, not the immediately-following trial, resulting in coding of that response as incorrect (nevertheless, still indicating that the fixation dimming was successfully detected). These high rates of detection and response suppression for fixation cross changes across participants and experiments indicate that participants were fixating correctly for the clear majority of the time.

Second, indirect evidence for the correct maintenance of fixation during the experiment comes from the simple effects of visual distractor position in the analysis. In the contrasts between left visual stimulation and right visual stimulation, all single subject and group analyses showed clear contralateral responses to the visual stimulation, indicating that the visual distractors were presented within the correct hemifield, and that visual fixation was reasonably constant throughout the experimental blocks (Supplementary Figure S3B).

**Supplementary References**

[S1] Shimojo S, Shams L (2001) Sensory modalities are not separate modalities: plasticity and interactions. Curr Opin Neurobiol 11: 505-509.

[S2] Laurienti PJ, Burdette JH, Wallace MT, Yen Y-F, Field AS, et al. (2002) Deactivation of sensory-specific cortex by cross-modal stimuli. J Cogn Neurosci 14: 420-429.

[S3] Raichle M, MacLeod AM, Snyder AZ, Powers WJ, Gusnard DA, et al. (2001) A default mode of brain function. Proc Natl Acad Sci USA 98: 676-682.

[S4] Eickhoff SB, Stephan KE, Mohlberg H, Grefkes C, Fink GR, et al. (2005) A new SPM toolbox for combining probabilistic cytoarchitectonic maps and functional imaging data. NeuroImage 25: 1325-1335.

#### Supplementary Figure Legends

**Figure S1**. Behavioural data. Data show the mean ± s.e.m. magnitude of multisensory integration effects (MSI, defined as performance on incongruent – congruent trials), across 13 participants per experiment (hand). Filled grey columns: visual distractor on the left of fixation. Open columns: visual distractor on the right of fixation. Left half of each panel: tool held in the left hand. Right half: tool held in the right hand. Left half of each of these sub-panels: tool tip positioned on the left of fixation. Right half: tool tip positioned on the right of fixation. **A.** RT. **B.** Errors.

**Figure S2**. Activity negatively correlated with multisensory integration (% error measures). Clusters of activation show predominantly right hemisphere brain areas in which the BOLD response significantly negatively covaried with the magnitude of multisensory integration across participants, overlaid on a standard MNI template brain. Voxels were thresholded at Z ≥ 2.33, p ≤ .01, and the resultant clusters were corrected for spatial extent across the whole brain, p≤.05. For display purposes the threshold was increased to Z ≥ 3.09, p ≤ .001. The data panels show percentage signal change against baseline (y-axis) against the magnitude of multisensory integration derived from percentage error measurements. For display purposes, data were pooled for the left hand (blue circles) and the right hand (red triangles). MSI: multisensory integration.

## **Figure S3**. Evidence that the participants maintained central visual fixation for the majority of the time during the experimental procedures. **A**. Percentage correct fixation task performance for the data of 13 participants in each experiment (Left hand tool use, Right hand tool use, along the x-axis). During the blocks of experimental trials, participants were required to monitor the fixation cross for brief (250ms) decreases in brightness. In response, participants were instructed to omit their response to the target on the subsequent trial. The broken horizontal line indicates chance performance at 8.33% correct. **B**. Simple effects of visual distractor side (VL>VR: left visual distractor>right visual distractor, cool colours; VR>VL: right visual distractor>left visual distractor, hot colours), for each participant in each experiment (L1-13: left hand tool use; R1-14: right hand tool use). Z-statistic contrast images were thresholded (Z ≥ 2.33, p ≤ .01, uncorrected), and overlaid on each participant’s anatomical scan in their native space. One slice (selected from the approximate MNI Z-coordinates +8 to +20) is shown for each participant, illustrating clusters of activation in occipital cortex.
