# Supplementary figures and images for: The Multisensory Attentional Consequences of Tool Use: A Functional Magnetic Resonance Imaging Study
Source: PLoS One. 2008 Oct 29;3(10):e3502. doi: 10.1371/journal.pone.0003502 (PMC2567039; doi:10.1371/journal.pone.0003502)

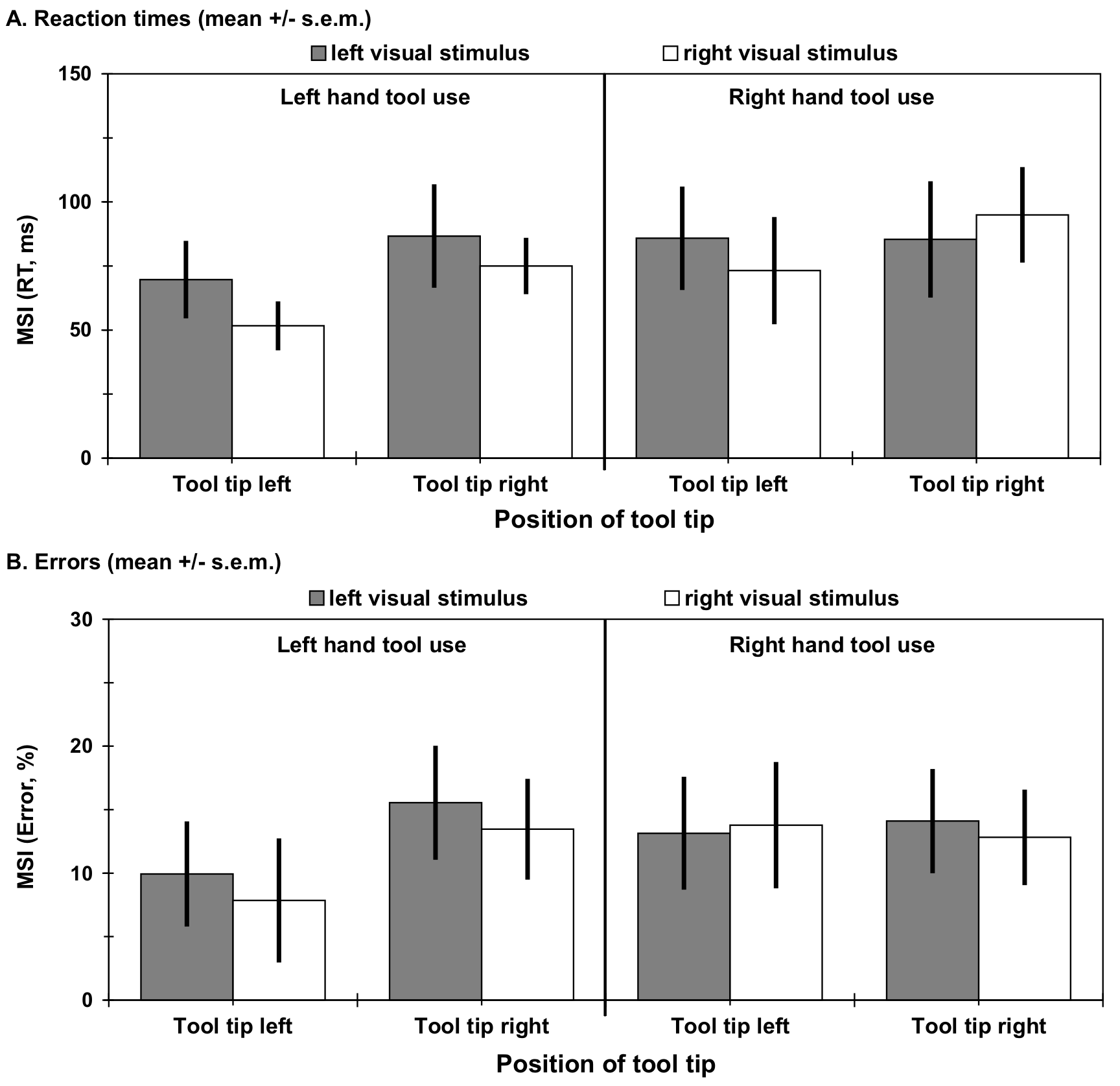

Supplement: Figure S1 — Behavioural data. Data show the mean±s.e.m. magnitude of multisensory integration effects (MSI, defined as performance on incongruent - congruent trials), across 13 participants per experiment (hand). Filled grey columns: visual distractor on the left of fixation. Open columns: visual distractor on the right of fixation. Left half of each panel: tool held in the left hand. Right half: tool held in the right hand. Left half of each of these sub-panels: tool tip positioned on the left of fixation. Right half: tool tip positioned on the right of fixation. A. RT. B. Errors. (0.16 MB TIF) [file pone.0003502.s002.tif]

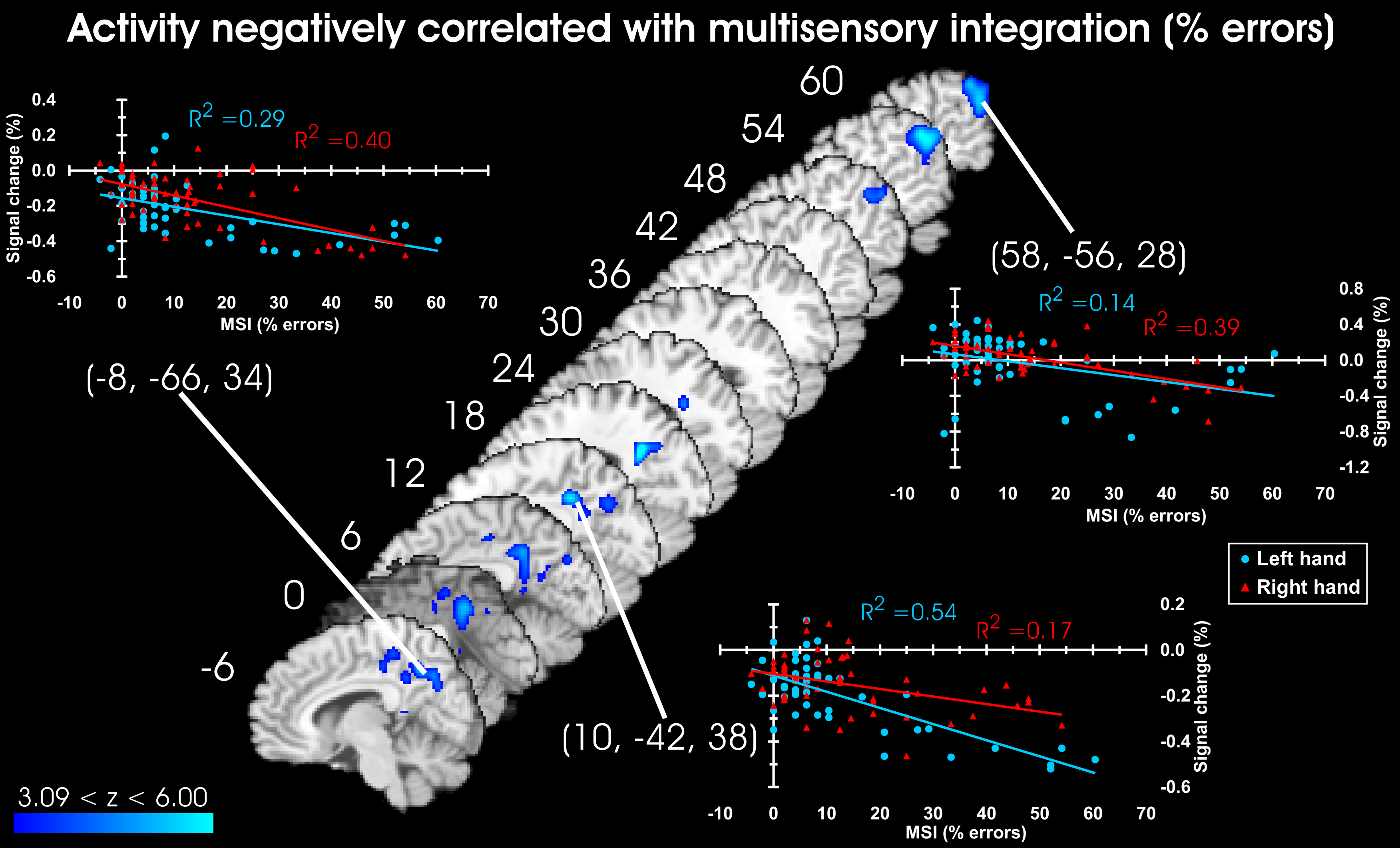

Supplement: Figure S2 — Activity negatively correlated with multisensory integration (% error measures). Clusters of activation show predominantly right hemisphere brain areas in which the BOLD response significantly negatively covaried with the magnitude of multisensory integration across participants, overlaid on a standard MNI template brain. Voxels were thresholded at ≥2.33, p≤.01, and the resultant clusters were corrected for spatial extent across the whole brain, p≤.05. For display purposes the threshold was increased to Z≥3.09, p≤.001. The data panels show percentage signal change against baseline (y-axis) against the magnitude of multisensory integration derived from percentage error measurements. For display purposes, data were pooled for the left hand (blue circles) and the right hand (red triangles). MSI: multisensory integration. (1.46 MB TIF) [file pone.0003502.s003.tif]

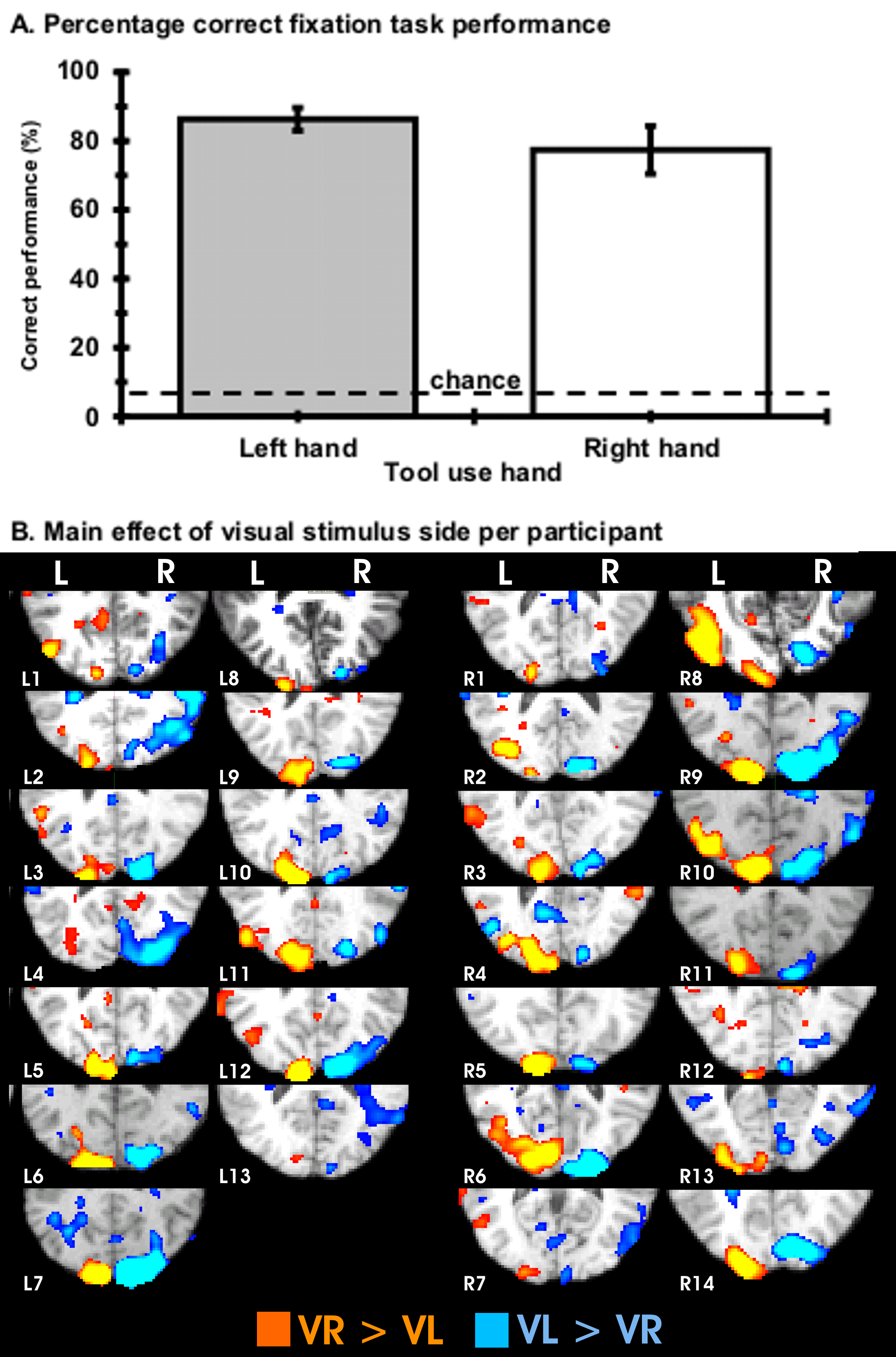

Supplement: Figure S3 — Evidence that the participants maintained central visual fixation for the majority of the time during the experimental procedures. A. Percentage correct fixation task performance for the data of 13 participants in each experiment (Left hand tool use, Right hand tool use, along the x-axis). During the blocks of experimental trials, participants were required to monitor the fixation cross for brief (250 ms) decreases in brightness. In response, participants were instructed to omit their response to the target on the subsequent trial. The broken horizontal line indicates chance performance at 8.33% correct. B. Simple effects of visual distractor side (VL>VR: left visual distractor>right visual distractor, cool colours; VR>VL: right visual distractor>left visual distractor, hot colours), for each participant in each experiment (L1–13: left hand tool use; R1–14: right hand tool use). Z-statistic contrast images were thresholded (Z≥2.33, p≤.01, uncorrected), and overlaid on each participant's anatomical scan in their native space. One slice (selected from the approximate MNI Z-coordinates +8 to +20) is shown for each participant, illustrating clusters of activation in occipital cortex. (2.65 MB TIF) [file pone.0003502.s004.tif]
