# Supplementary material for: The Multisensory Attentional Consequences of Tool Use: A Functional Magnetic Resonance Imaging Study
Source: PLoS One. 2008 Oct 29;3(10):e3502. doi: 10.1371/journal.pone.0003502 (PMC2567039; doi:10.1371/journal.pone.0003502)
Supplement: Table S1 — (0.05 MB DOC) [file pone.0003502.s005.doc]

Table S1. Simple effects of visual distractor position and tool tip position

|  |  |  |  |  | **Peak voxel Z-statistics** | | | | | | |
| --- | --- | --- | --- | --- | --- | --- | --- | --- | --- | --- | --- |
|  |  |  |  | **Peak voxel coordinates (mm)** | **Interactions** | | **Simple tool-position effects** | | **Visual effects** | **Multisensory** | |
| **Fig.** | **Hem.** | **Gyrus/ Sulcus** | **BA** | **MNI152** | **TxV** | **HxTxV** | **TLVL> TRVL** | **TRVR> TLVR** | **L>R** | RT | Error |
| Contralateral tool-position-dependent increases in BOLD response | | | | | | | | | | | |
|  | R | pIPS | 7/39 | (32, -80, 40) | 1.74 | 0.31 | **2.50*** | -0.07 | **3.42**** | 2.15 | 1.03 |
|  | R | MOG | 19/37 | (50, -78, 16) | 0.30 | -0.66 | **2.34*** | -1.94 | **4.16***** | 0.56 | 2.14 |
| 2A | L | CalS | 17‡ | (-14, -86, 12) | 2.27 | -1.60 | 0.32 | **2.87*** | **-3.80***** | -0.43 | -1.16 |
| 2B | R | LiG | 18 | (20, -68, -2) | 0.88 | -2.21 | **3.55**** | **-2.41*** | **5.44***** | **3.20**** | **2.95*** |
| Ipsilateral tool-position-dependent decreases in BOLD response | | | | | | | | | | | |
| 2C | R | SOG | 19 | (20, -86, 18) | -1.75 | -0.22 | 1.22 | **-3.57**** | **6.48***** | 1.03 | 1.19 |
| 2D | R | IOG | 19 | (44, -84, -6) | -1.01 | -0.55 | 1.28 | **-2.66*** | **4.98***** | 1.39 | **3.26**** |
|  | R | Occipital Pole | 17/18‡ | (14, -92, -8) | -1.28 | -1.06 | 0.56 | **-2.34*** | **6.30***** | **2.46*** | 1.56 |

Fig.: Figure showing percent signal change for this area. Hem.: Hemisphere. BA: Probable Brodmann’s area (*according to probabilistic cytoarchitecture maps where available: Voxel has ≥30% probability of being assigned to that area, and a total of ≥50% probability of being assigned to any area(s), [S4]). MNI152: Montreal Neurological Institute standard brain coordinates (average of 152 brains). T: Tool tip position; V: Visual distractor position; L: Left; R: Right; TxV: [(TLVL+TRVR)>(TLVR + TRVL)]. HxTxV: Left hand ([(TLVL+TRVR)>(TLVR + TRVL)]) – Right hand([(TLVL+TRVR)–(TLVR + TRVL)]). Visual effects: ±[(TLVL+TRVL)>(TLVR+TRVR)]. RT: Multisensory integration in reaction time measures; Error: Multisensory integration in error measures. *: p≤.01; **: p≤.001; ***: p≤.0001, voxelwise uncorrected. Criteria for inclusion in above table were: 1) A peak voxel of Z≥2.33 in either significant visual contrast; 2) The same voxel has Z≥2.33 for either TLVL>TRVL or TRVR>TLVR, or the reversed contrasts; 3) The percent signal change for the voxel and several neighbouring voxels showed no significant block-order confound main effects or interactions (p>.01). pIPS: Posterior intra-parietal sulcus. MOG: Middle occipital gyrus. CalS: Calcarine sulcus. LiG: Lingual gyrus. SOG: Superior occipital gyrus. IOG: Inferior occipital gyrus.
