# Supplementary material for: The Multisensory Attentional Consequences of Tool Use: A Functional Magnetic Resonance Imaging Study
Source: PLoS One. 2008 Oct 29;3(10):e3502. doi: 10.1371/journal.pone.0003502 (PMC2567039; doi:10.1371/journal.pone.0003502)
Supplement: Table S2 — (0.05 MB DOC) [file pone.0003502.s006.doc]

Table S2. Interaction between visual distractor position and tool tip position

|  |  |  |  | **Peak voxel Z-statistics** | | | | | | |
| --- | --- | --- | --- | --- | --- | --- | --- | --- | --- | --- |
|  |  |  | **Peak voxel coordinates (mm)** | **Interactions** | | **Simple tool-position effects** | | **Visual effects** | **Multisensory** | |
| **Hem.** | **Gyrus/**  **Sulcus** | **BA** | **MNI152** | **TxV** | **HxTxV** | **TLVL> TRVL** | **TRVR> TLVR** | **L>R** | RT | Error |
| Positive hand non-specific effects of tool position ([tool & visual distractor on same side] > [on opposite sides]) | | | | | | | | | | |
| L | SOG/AG | 19/39 | (-38, -64, 24) | **3.18**** | 0.78 | **3.43**** | 1.07 | 0.51 | -1.0 | -1.48 |
| R | SOG/AG | 7/39/40 | (36, -58, 44) | **2.95*** | -1.37 | 1.98 | 2.25 | -0.65 | 0.18 | -0.11 |
| Negative hand non-specific effects of tool position ([tool & visual distractor on opposite sides] > [on same side]) | | | | | | | | | | |
|  | Precun. | 5/7 | (0, -58, 52) | **-3.03*** | 1.08 | **-2.61*** | -1.71 | -0.62 | 0.08 | 0.36 |
| R | SFS/MFG | 8 | (30, 12, 60) | **-2.42*** | 1.87 | -1.25 | -2.20 | 1.10 | **4.63***** | 1.83 |
| Positive hand-specific effects of tool position (as above, but greater for left hand than for right hand tool use) | | | | | | | | | | |
| L | SOG/AG | 39 | (-42, -66, 32) | **2.33*** | **2.36*** | **2.83*** | 0.45 | 1.71 | -0.92 | -0.46 |
| L | SMG/ AG | 40/39 | (-54, -50, 50) | 1.77 | **2.83*** | 0.22 | 2.27 | -0.96 | 0.46 | 0.54 |
| Negative hand-specific effects of tool position (as above, but greater for right hand than for left hand tool use) | | | | | | | | | | |
| L | SOG | 19 | (-30, -88, 24) | -0.16 | **-2.84*** | -0.13 | -0.52 | **-2.66*** | -0.61 | 0.75 |
| L | MOG | 19 | (-48, -78, 18) | -0.29 | **-2.49*** | -0.73 | 0.31 | **-2.67*** | -0.64 | -0.97 |
| R | Precentral gyrus/IFG | 44‡ | (52, 8, 8) | -0.11 | **-2.84*** | 0.71 | -0.86 | 1.53 | 1.63 | 1.35 |

Hem.: Hemisphere. BA: Probable Brodmann’s area (‡according to probabilistic cytoarchitecture maps where available: Voxel has ≥30% probability of being assigned to that area, and a total of ≥50% probability of being assigned to any area(s), [S4]). MNI152: Montreal Neurological Institute standard brain coordinates (average of 152 brains). T: Tool tip position; V: Visual distractor position; L: Left; R: Right; TxV: [(TLVL+TRVR)>(TLVR + TRVL)]. HxTxV: Left hand ([(TLVL+TRVR)>(TLVR + TRVL)]) – Right hand([(TLVL+TRVR)–(TLVR + TRVL)]). Visual effects: ±[(TLVL+TRVL)>(TLVR+TRVR)]. RT: Multisensory integration in reaction time measures. Error: Multisensory integration in error measures. *: p≤.01; **: p≤.001; ***: p≤.0001, voxelwise uncorrected. Criteria for inclusion in above table were: 1) A peak voxel of Z≥2.33 approximately in BA5, 6, 7, 8, 44, or 45, for the interactions, TxV and HxTxV; 2) The same voxel has Z≥2.33 for either TLVL>TRVL or TRVR>TLVR, or the reversed contrasts; 3) The percent signal change for the voxel and several neighbouring voxels showed no significant block-order confound main effects or interactions (p>.01). SOG: Superior occipital gyrus. AG: Angular gyrus. Precun.: Precuneus. SFS: Superior frontal sulcus. MFG: Middle frontal gyrus. SMG: Supramarginal gyrus. MOG: Middle occipital gyrus. IFG: Inferior frontal gyrus.
