# Supplementary material for: The Multisensory Attentional Consequences of Tool Use: A Functional Magnetic Resonance Imaging Study
Source: PLoS One. 2008 Oct 29;3(10):e3502. doi: 10.1371/journal.pone.0003502 (PMC2567039; doi:10.1371/journal.pone.0003502)
Supplement: Table S3 — (0.06 MB DOC) [file pone.0003502.s007.doc]

Table S3. Positive covariation between BOLD response and multisensory integration

|  |  |  |  |  | **Peak voxel Z-statistics** | | | | | | |
| --- | --- | --- | --- | --- | --- | --- | --- | --- | --- | --- | --- |
|  |  |  |  | **Peak voxel coordinates (mm)** | **Interactions** | | **Simple tool-position effects** | | **Visual effects** | **Multisensory** | |
| **Fig.** | **Hem.** | **Gyrus/ Sulcus** | **BA** | **MNI152** | **TxV** | **HxTxV** | **TLVL> TRVL** | **TRVR> TLVR** | **L>R** | RT | Error |
| Positive correlation with reaction time congruency effects | | | | | | | | | | | |
| 3A | R | MFG | 8/6 | (32, 10, 62) | -2.07 | 1.18 | -1.17 | -1.77 | 1.19 | **5.48***** | **2.94*** |
|  | R | mSFG | 8 | (4, 32, 48) | 0.87 | -0.08 | 0.83 | 0.40 | -0.01 | **4.59***** | **3.44**** |
|  | R | SFG | 8/6‡ | (12, 24, 60) | -0.85 | 0.40 | -0.65 | -0.56 | 0.33 | **4.40***** | **2.51*** |
|  | R | SMA/ACG | 6‡/24 | (8, 0, 46) | -0.11 | -0.63 | 0.32 | -0.48 | 0.01 | **3.51**** | 1.13 |
| Positive correlation with percentage error congruency effects | | | | | | | | | | | |
| Cluster 1 | | | | | | | | | | | |
| 3B | R | CB(ve.) |  | (2, -64, -14) | -0.83 | 0.53 | -0.89 | -0.28 | -0.05 | 1.83 | **5.47***** |
|  | R | CB(ve.) |  | (8, -52, -6) | 0.32 | 0.32 | 0.62 | -0.17 | 0.37 | 2.26 | **6.78***** |
|  | R | CB |  | (18, -36, -22) | -1.02 | -0.23 | -0.89 | -0.56 | -0.74 | **2.66*** | **4.35***** |
|  | R | CB/FuG | 19 | (32, -60, -20) | -0.21 | -0.53 | 0.19 | -0.48 | **3.34**** | **2.72*** | **4.47***** |
| Cluster 2 | | | | | | | | | | | |
| 3B | L | IOG/MTG | 19/37 | (-52, -74, 0) | 0.47 | -1.18 | -0.50 | 1.17 | **-3.51**** | 1.35 | **3.93***** |
|  | L | MOG | 18/19 | (-32, -90, 10) | 0.46 | -0.70 | 0.69 | -0.04 | **-3.02*** | **2.67*** | **5.18***** |
|  | L | MOG/SOG | 18‡/17‡ | (-18, -98, 10) | -0.26 | 0.21 | -0.69 | 0.33 | **-5.55***** | **2.45*** | **4.84***** |
| Cluster 3 | | | | | | | | | | | |
| 3B | R | MOG/IOG | 18/19 | (42, -90, 4) | 0.57 | -1.40 | 1.74 | -0.95 | **4.64***** | 2.11 | **4.81***** |
|  | R | Precun. | 7/5 | (10, -58, 68) | -1.02 | -0.00 | -2.06 | 0.64 | -1.00 | **2.89*** | **6.37***** |
|  | R | POS | 19/7 | (28, -70, 26) | 0.93 | -0.02 | 1.62 | -0.33 | 1.32 | **3.69**** | **4.66***** |
| Cluster 4 | | | | | | | | | | | |
| 3B |  | mSFG | 8 | (0, 30, 46) | 0.60 | -0.11 | 0.07 | 0.78 | -0.34 | **3.95***** | **4.33***** |
|  | R | mSFG | 6‡/8 | (4, 22, 56) | 0.41 | 0.55 | -0.29 | 0.86 | 0.23 | **3.66**** | **4.69***** |
|  | R | SFS | 6 | (28, -2, 56) | 0.01 | 1.09 | 0.79 | -0.78 | 1.49 | **2.47*** | **3.63**** |

Fig.: Figure showing percent signal change for this area. Hem.: Hemisphere. BA: Probable Brodmann’s area (‡according to probabilistic cytoarchitecture maps where available: Voxel has ≥30% probability of being assigned to that area, and a total of ≥50% probability of being assigned to any area(s), [S4]). MNI152: Montreal Neurological Institute standard brain coordinates (average of 152 brains). T: Tool tip position; V: Visual distractor position; L: Left; R: Right; TxV: [(TLVL+TRVR)>(TLVR + TRVL)]. HxTxV: Left hand ([(TLVL+TRVR)>(TLVR + TRVL)]) – Right hand([(TLVL+TRVR)–(TLVR + TRVL)]). Visual effects: ±[(TLVL+TRVL)>(TLVR+TRVR)]. RT: Multisensory integration in reaction time measures; Error: Multisensory integration in error measures. *: p≤.01; **: p≤.001; ***: p≤.0001, voxelwise uncorrected. Criteria for inclusion in above table were: 1) A peak voxel of Z≥2.33 in either significant RT or error (positive covariation) multisensory contrast; 2) The percent signal change for the voxel and several neighbouring voxels showed no significant block-order confound main effects or interactions (p>.01). MFG: Middle frontal gyrus. mSFG: Medial superior frontal gyrus. SFG: Superior frontal gyrus. SMA: Supplementary motor area. ACG: Anterior cingulate gyrus. CB(ve.): Cerebellar vermis. CB: Cerebellum. FuG: Fusiform gyrus. IOG: Inferior occipital gyrus. MTG: Middle temporal gyrus. MOG: Middle occipital gyrus. SOG: Superior occipital gyrus. Precun.: Precuneus. POS: Parietal-occipital sulcus. SFS: Superior frontal sulcus.
