# Supplementary material for: The Multisensory Attentional Consequences of Tool Use: A Functional Magnetic Resonance Imaging Study
Source: PLoS One. 2008 Oct 29;3(10):e3502. doi: 10.1371/journal.pone.0003502 (PMC2567039; doi:10.1371/journal.pone.0003502)
Supplement: Table S4 — (0.05 MB DOC) [file pone.0003502.s008.doc]

**Table S4. Negative covariation between BOLD response and multisensory integration**

|  |  |  |  |  | **Peak voxel Z-statistics** | | | | | | |
| --- | --- | --- | --- | --- | --- | --- | --- | --- | --- | --- | --- |
|  |  |  |  | **Peak voxel coordinates (mm)** | **Interactions** | | **Simple tool-position effects** | | **Visual effects** | Multisensory | |
| **Fig.** | **Hem.** | **Gyrus/ Sulcus** | **BA** | **MNI152** | **TxV** | **HxTxV** | **TLVL> TRVL** | **TRVR> TLVR** | **L>R** | RT | Error |
| Cluster 1 | | | | | | | | | | | |
| S2 | R | AG/SMG/STG | 39/40/22 | (58, -56, 28) | -0.06 | 0.19 | 0.74 | 1.21 | -1.29 | **-2.85*** | **-5.34***** |
|  | R | AG/SMG | 39/40 | (56, -50, 38) | 1.18 | 0.55 | 1.01 | 0.66 | 0.50 | **2.62*** | **-5.60***** |
| Cluster 2 | | | | | | | | | | | |
| S2 | R | Precun./pCiG | 7/19/ 23 | (18, -54, 32) | 0.29 | 1.08 | 0.36 | 0.04 | 0.47 | **-4.11***** | **-6.20***** |
|  | R | POS/Precun. | 7/19 | (16, -66, 34) | -0.78 | 1.13 | -0.15 | -0.95 | 1.04 | **-3.31**** | **-4.52***** |
|  | R | pCiG | 23 | (10, -42, 38) | -0.01 | -0.04 | 0.15 | -0.16 | 0.06 | **-3.45**** | **-5.45***** |
|  | R | POS/Cun. | 18/19 | (8, -80, 34) | -1.02 | -1.02 | -0.40 | -1.04 | 1.26 | **-2.93*** | **-3.77***** |
|  | R | pCiG/Precun. | 19/23 | (4, -46, 10) | -0.25 | 0.03 | 0.09 | -0.45 | 0.45 | -1.63 | **-3.41**** |
|  | L | Precun./pCiG | 7/23 | (-4, -56, 40) | -0.77 | 0.42 | 0.17 | -1.25 | -0.92 | **-3.04*** | **-5.01***** |
|  | L | PreCG/pCiG | 4a‡/ 23 | (-4, -36, 54) | -0.02 | 0.34 | -0.03 | 0.01 | -0.42 | **-3.19**** | **-3.93***** |
|  | L | pCiG | 23 | (-6, -28, 44) | -0.15 | 0.33 | -0.57 | 0.36 | -0.87 | **-2.61*** | **-3.78***** |
|  | L | POS/Precun. | 7 | (-8, -66, 34) | -0.00 | -0.25 | 0.45 | 0.51 | -0.51 | **-2.45*** | **-4.36***** |
|  | L | Thal. |  | (-16, -28, 4) | 0.24 | -0.50 | 0.34 | -0.00 | -0.41 | **-2.01** | **-4.11***** |

Fig.: Figure showing percent signal change for this area. Hem.: Hemisphere. BA: Probable Brodmann’s area (‡according to probabilistic cytoarchitecture maps where available: Voxel has ≥30% probability of being assigned to that area, and a total of ≥50% probability of being assigned to any area(s), [S4]). MNI152: Montreal Neurological Institute standard brain coordinates (average of 152 brains). T: Tool tip position; V: Visual distractor position; L: Left; R: Right; TxV: [(TLVL+TRVR)>(TLVR + TRVL)]. HxTxV: Left hand ([(TLVL+TRVR)>(TLVR + TRVL)]) – Right hand([(TLVL+TRVR)–(TLVR + TRVL)]). Visual effects: ±[(TLVL+TRVL)>(TLVR+TRVR)]. RT: Multisensory integration in reaction time measures; Error: Multisensory integration in error measures. *: p≤.01; **: p≤.001; ***: p≤.0001, voxelwise uncorrected. Criteria for inclusion in above table were: 1) A peak voxel of Z≥2.33 in significant error (negative covariation) multisensory contrast; 2) The percent signal change for the voxel and several neighbouring voxels showed no significant block-order confound main effects or interactions (p>.01). AG: Angular gyrus. SMG: Supramarginal gyrus. STG: Superior temporal gyrus. Precun.: Precuneus. pCiG: Posterior cingulate gyrus. POS: Parietal-occipital sulcus. Cun.: Cuneus. LiG: Lingual gyrus. Thal.: Thalamus.
